# An Evolution-Guided Analysis Reveals a Multi-Signaling Regulation of Fas by Tyrosine Phosphorylation and its Implication in Human Cancers

**DOI:** 10.1371/journal.pbio.1002401

**Published:** 2016-03-04

**Authors:** Krittalak Chakrabandhu, Sébastien Huault, Jérôme Durivault, Kévin Lang, Ly Ta Ngoc, Angelique Bole, Eszter Doma, Benoit Dérijard, Jean-Pierre Gérard, Michel Pierres, Anne-Odile Hueber

**Affiliations:** 1 Univ. Nice Sophia Antipolis, CNRS, Inserm, iBV, 06100 Nice, France; 2 Centre d'Immunologie de Marseille-Luminy, Aix-Marseille Université, UM2, Marseille, France, INSERM, U1104, Marseille, France, and CNRS, UMR 7280, Marseille, France; 3 Service de radiothérapie, Centre Antoine Lacassagne, Nice, France; St. Jude Children's Research Hospital, UNITED STATES

## Abstract

Demonstrations of both pro-apoptotic and pro-survival abilities of Fas (TNFRSF6/CD95/APO-1) have led to a shift from the exclusive “Fas apoptosis” to “Fas multisignals” paradigm and the acceptance that Fas-related therapies face a major challenge, as it remains unclear what determines the mode of Fas signaling. Through protein evolution analysis, which reveals unconventional substitutions of Fas tyrosine during divergent evolution, evolution-guided tyrosine-phosphorylated Fas proxy, and site-specific phosphorylation detection, we show that the Fas signaling outcome is determined by the tyrosine phosphorylation status of its death domain. The phosphorylation dominantly turns off the Fas-mediated apoptotic signal, while turning on the pro-survival signal. We show that while phosphorylations at Y232 and Y291 share some common functions, their contributions to Fas signaling differ at several levels. The findings that Fas tyrosine phosphorylation is regulated by Src family kinases (SFKs) and the phosphatase SHP-1 and that Y291 phosphorylation primes clathrin-dependent Fas endocytosis, which contributes to Fas pro-survival signaling, reveals for the first time the mechanistic link between SFK/SHP-1-dependent Fas tyrosine phosphorylation, internalization route, and signaling choice. We also demonstrate that levels of phosphorylated Y232 and Y291 differ among human cancer types and differentially respond to anticancer therapy, suggesting context-dependent involvement of Fas phosphorylation in cancer. This report provides a new insight into the control of TNF receptor multisignaling by receptor phosphorylation and its implication in cancer biology, which brings us a step closer to overcoming the challenge in handling Fas signaling in treatments of cancer as well as other pathologies such as autoimmune and degenerative diseases.

## Abstract 2

Signalling by the tumor necrosis factor receptor (TNFR) superfamily member Fas can promote either survival or death of a cell, but the mechanism underlying this choice is unclear. This study reveals that the outcome of Fas signalling (death versus survival) is determined by the tyrosine phosphorylation status of its death domain.

## Author Summary

The versatility of the tumor necrosis factor receptor superfamily members in cell fate regulation is well illustrated by the dual signaling generated by one of the most extensively studied members of the family, Fas (CD95/TNFSFR6). Upon binding its ligand, Fas is able to elicit both pro-death and pro-survival signals. Until now, we have lacked mechanistic knowledge about when and how one signaling output of Fas is favored over the other. We demonstrate here that the outcome of Fas signaling is determined by the phosphorylation status of two tyrosine residues (Y232 and Y291) within the death domain. Dephosphorylation of Fas tyrosines by SHP-1 tyrosine phosphatase turns on the pro-apoptotic signal whereas the tyrosine phosphorylation by Src family kinases (SFKs) turns off the pro-apoptotic signal and turns on the pro-survival signal. Furthermore, we provide evidence that Fas tyrosine phosphorylation status may vary among different cancer types and influence the response to anti-cancer treatments. This information reveals an opportunity to use the screening of Fas tyrosine phosphorylation, a newly discovered direct molecular indicator of Fas functional output, to aid the design of Fas-related cancer therapies.

## Introduction

Fas (CD95/APO-1/TNFRSF6), a tumor necrosis factor (TNF) receptor superfamily member, is a well-known apoptosis activator. The binding with Fas ligand (FasL) can lead to the recruitment of Fas-associated protein with death domain (FADD) and procaspase-8, forming the death-inducing signaling complex (DISC). This results in the activation of the caspase cascade and, ultimately, apoptosis [1]. Fas was mainly considered as a tumor suppressor thanks to its familiar ability to promote programmed cell death (apoptosis). However, accumulating evidence supports a significant role of Fas in the alternative non-death signaling leading to cell survival, proliferation, motility, epithelial-mesenchymal transition, cancer growth, and metastasis in some contexts [2]. While such conditional multisignaling of Fas has also been well demonstrated in several cancer models, including colon cancer [3–5], the mechanism controlling these multisignals is unclear.

Fas multiple signaling implies an efficient molecular switch mechanism that lends itself to a flexible formation of different signaling complexes depending on the type of signal being transmitted. One of such mechanisms to be considered is tyrosine phosphorylation. While it has been shown for almost two decades that both tyrosines in the intracellular domain, Y232 and Y291, can be phosphorylated [6], the role of their phosphorylation is not understood. Due to the lack of tools for functional analysis and site-specific phospho-tyrosine (pY) detection, there have been only few conflicting reports that infer functions of Fas phosphorylation [7–8] and, thus, limiting our understanding of the functions of each pY in Fas signaling.

Taking a unique approach based on Fas protein evolution analysis, evolution-guided Fas pY proxy, and site-specific detection of Fas pY, we show for the first time that Fas death domain tyrosine phosphorylation is a dominant anti-apoptosis and a pro-survival mechanism. We discover that while phosphorylations at Y232 and Y291 share the anti-apoptotic function, they differ in terms of structural requirement and other functions. We also reveal the regulation of Fas pY by SFK/SHP-1-based system and the functions of death domain pY in the control of DISC formation and clathrin-dependent Fas endocytosis. Furthermore, we present the implication of the pY-based regulation of Fas signaling in different human cancers along with potential means to predict Fas signaling modes, which is crucial for Fas-related therapeutic design to achieve clinical success.

## Results

### Evolution-Guided Analysis and Site-Directed Mutagenesis Demonstrate that Death Domain Tyrosine Phosphorylation Is Dispensable for Fas-Induced Apoptosis

**Fig 1:**
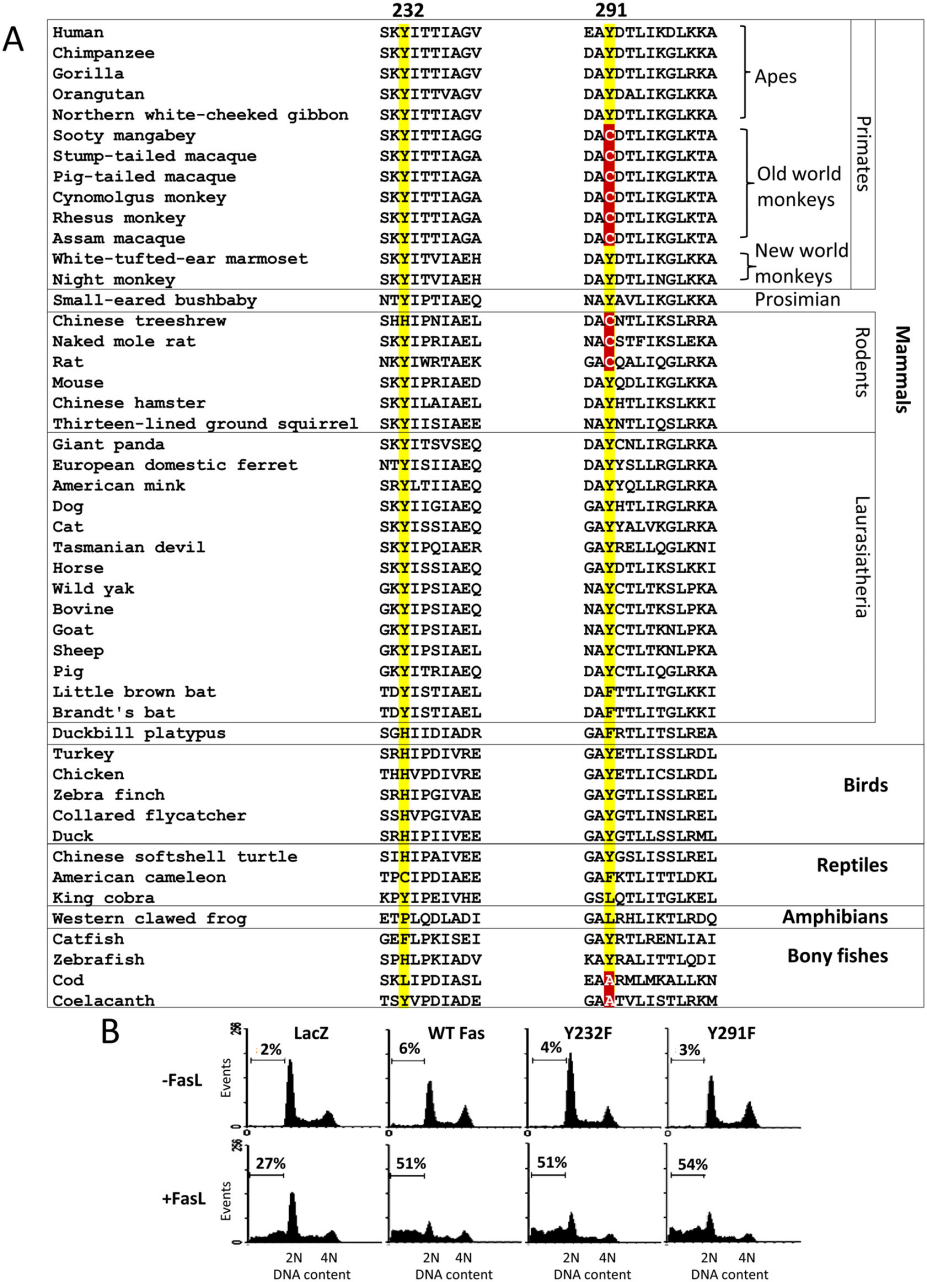
A common cysteine substitution for tyrosine in the Fas death domain in primates and rodents and site-directed mutagenesis suggest that the tyrosine phosphorylation is dispensable for Fas apoptosis. (A) Partial sequence alignment of Fas death domain (helices 1 and 5 [9]) in vertebrates. Positions 232 and 291 of human Fas are highlighted. Cysteine and alanine at the 291 position of Fas are boxed in red. (B) SW480 cells stably expressing control protein (LacZ.V5) or indicated Fas.V5 proteins were treated with 10 ng/ml FasL crosslinked with anti-FLAG (M2) for 24 h and subsequently subjected to DNA content analysis by flow cytometry. Numbers indicate percentage of cells that underwent apoptosis, having subG1 DNA content due to DNA fragmentation.

To date, the active role of each pY of the Fas death domain in apoptosis induction by FasL in the human cell system is unknown. To approach this issue, we turned toward the evolution of Fas protein as a guide by considering the substitutions of amino acids at these phosphorylation sites during the course of evolution. Multiple sequence alignment of Fas proteins from vertebrates illustrates that the side chain size and aromatic ring feature are highly conserved at position 232. This, however, is not the case for position 291, where neither the size nor the aromatic side chain of tyrosine is a substitution criterion (Fig 1A; positions Y232 and Y291 of human Fas are used as references to indicate corresponding amino acid positions in other species throughout the text). Notably, substitution of Y by a small amino acid, cysteine (C), is common among primates (particularly in old world monkeys) and rodents, which are relatively close to hominoids (apes, including human). Further in evolutional distance, one can also observe the substitution of Y291 by a small amino acid, alanine (A), in some fishes, including coelacanth (the living fossil) and cod.

Small amino acid substitutions for Y291 in closely related species do not appear to impact the apoptotic functions of Fas. Previous work has shown that, like in human and mouse, Fas in cynomolgus monkey and rat that carries a C at position 291 could signal apoptosis upon ligation with an agonistic antibody [10] and FasL [8,11], respectively. The observation that Y at the position 291 is interchangeable with C among closely related species whose Fas can function as an apoptosis inducer suggests that the presence of Y at this position and, thus, its phosphorylation is not essential for Fas apoptotic signal. Our observation that in several human cell types unphosphorylated mutants (Y232F and Y291F) could transmit apoptotic signals supports this conclusion (Figs 1B, S1 and S2).

### Evolution-Guided, Site-Directed Mutagenesis Reveals the Anti-Apoptotic Role of Death Domain Tyrosine Phosphorylation

The above-mentioned results led us to hypothesize that the advantage of Fas death domain pY, if it occurred through evolution, was to provide a reversible switch from the apoptotic signal to other signals, e.g., the survival signal. However, to clarify whether Fas pY plays active roles in cellular processes, an ability to induce and maintain, or mimic the properties of, the phosphorylated state of amino acid residues of interest in cells is required.

A comparative genomic study of Raf kinases shows that pY could have evolved from smaller acidic amino acids such as aspartic acid (D) or glutamic acid (E) [12]. Thus, substituting D or E for pY may mimic the phosphorylated state of some proteins [13–16]. However, such substitutions require careful consideration to ensure that the observed results are not due to the change in the amino acid size.

The common substitution between small amino acids and Y at position 291, but not at position 232, of Fas in vertebrates suggests that, depending on the sites, net charge can be more important than the details of the side chain structure. To investigate this issue, we performed evolution-guided, site-directed mutagenesis to examine the functional effects of the following amino acid substitutions on Fas: 1. Size reduction by (a) substituting Y232 with C, as observed in reptile and in cases of human autoimmune lymphoproliferative syndrome (ALPS) [17], (b) substituting Y291 with A and C, as observed in fish and mammals respectively; and 2. negative charge addition by substituting Y232 and Y291 with the acidic D, which has a size comparable to C, the most common small amino acid substituting for Y in evolution of Fas. The features of amino acids used in the site-directed mutagenesis are summarized in S3 Fig.

**Fig 2:**
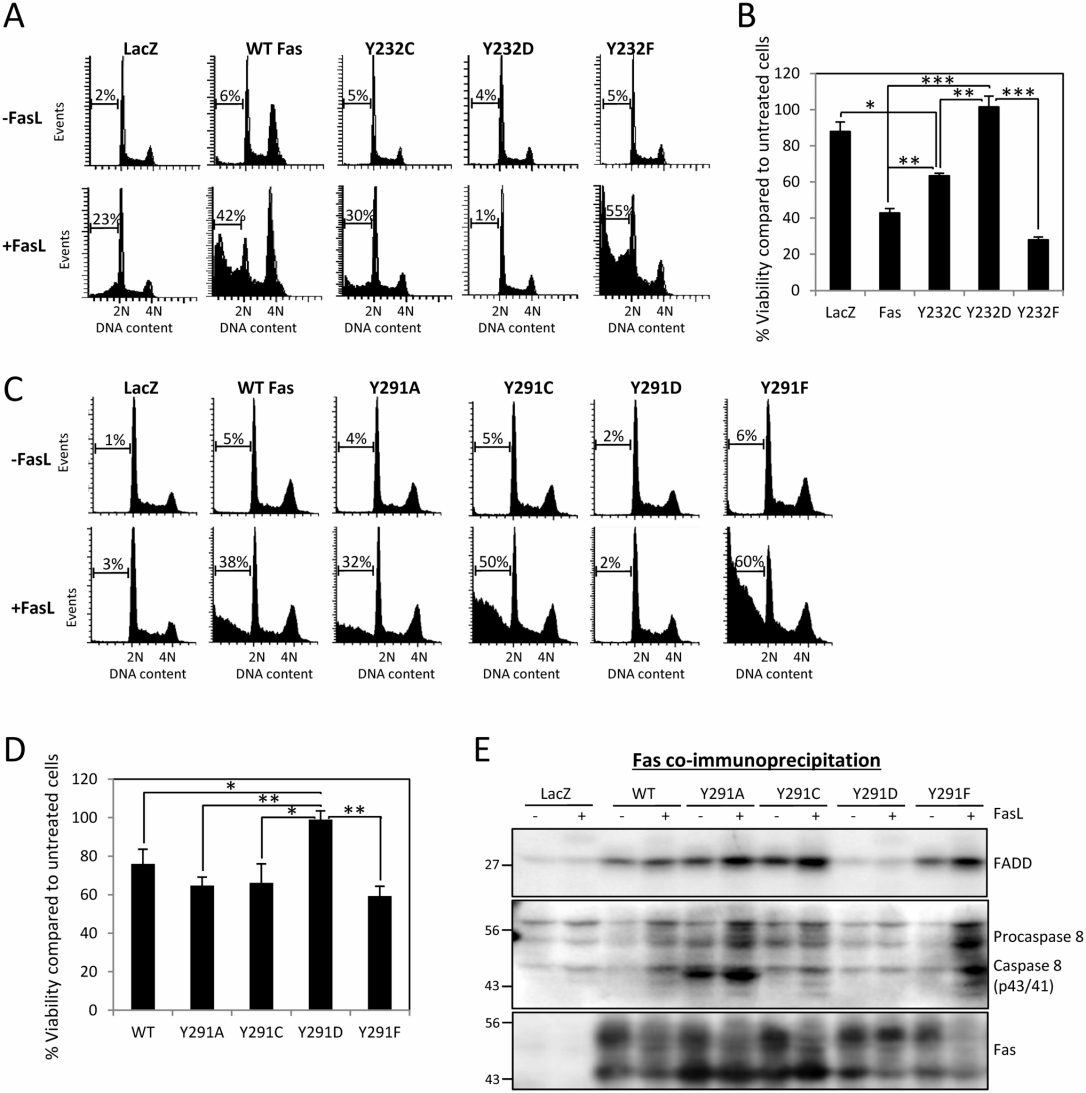
The charge of the 291 residue is more important for the function of Fas than the size and details of the side chain. (A) SW480 cells stably expressing control protein (LacZ.V5), V5-tagged wild type Fas, or indicated Y232 phosphorylation mutants were treated with 50 ng/ml FasL crosslinked with 1 μg/ml M2 for 4 h and analysed by flow cytometry. Numbers indicated percentage of cells that underwent apoptosis, having subG1 DNA content due to DNA fragmentation. (B) Cells, as in (A), were treated with 20 ng/ml FasL crosslinked with 1 μg/ml M2 for 24h. The cell viability was measured by WST-1 assay and presented as percentage of cell viability compared to untreated control cells. Values represent means ± SEM from three independent experiments (* *p* < 0.05, ** *p* < 0.01, *** *p* < 0.001, unpaired *t* test). (C) SW480 cells stably expressing LacZ.V5, V5-tagged wild-type Fas, or indicated Y291 phosphorylation mutants were treated with 10 ng/ml FasL crosslinked with 1 μg/ml M2 for 4 h and analysed for apoptosis as in (A). (D) Cells, as in (C), were treated with 10 ng/ml FasL crosslinked with M2 for 24 h. The cell viability was measured by WST-1 assay as in (B). Values represent means ± SEM from three independent experiments (* *p* < 0.05, ** *p* < 0.01, *** *p* < 0.001, unpaired *t* test). (E) Cells, as in (C), were treated with 10 ng/ml FasL crosslinked with 1 μg/ml M2 or untreated (control) for 10 min. The lysates were collected and subjected to coimmunoprecipitation with anti-Fas antibody followed by SDS-PAGE and immunoblotting with indicated antibodies. Note that the low level of immunoprecipitated Fas from the LacZ sample could be visualized on an image from long exposure. Equivalent Fas expression levels in different stable cell lines are shown in S4 Fig. Numerical values underlying the data summary displayed in this figure can be found in S1 Data.

We observed that introducing a negative charge by Y232D mutation rescued cells from FasL-induced cell death (Fig 2A and 2B). However, side chain size reduction by Y232C mutation also, to a lesser extent, rescued the cells, suggesting the importance of side chain size and aromatic ring of Y232 in Fas signaling. This is in accord with the high conservation of aromatic amino acids at this position in vertebrates (Fig 1A). Since the complete rescue observed in Y232D-carrying cells could be a combined effect of the added negative charge and reduced side chain size, substituting a small acidic amino acid for pY (as a single measure) may not provide an adequate proxy for functional studies of phospho-Y232 (pY232).

However, the situation differed for the 291 position where, similar to Y291F, size-reduction substitutions (Y291A and Y291C) had no impact on the apoptotic function (Fig 2C and 2D) and the formation of the DISC of Fas (Fig 2E). In contrast, the negative-charge substitution, Y291D, completely abolished FasL-induced apoptosis and DISC formation (Fig 2C and 2E). This indicates that the abolition of Fas apoptotic signaling was due to the negative charge of aspartic acid but not to its small size. Of note is that while Y291A mutation did not impact the apoptotic function of Fas, we observed an increase in cleaved fragments of caspase 8 in the DISC from stimulated Y291A cell lysate. This phenomenon did not cause any spontaneous cell death (Fig 2C) and could be related to non-apoptotic function of caspase 8, as it is now well-known that the death-effector domains (DEDs) containing proteins, including caspases, not only regulate apoptosis but also other forms of cell death, including necroptosis, as well as other important cellular processes such as autophagy and inflammation (see review [18]). Further studies are required to explore the roles and effects of basal activation of caspase 8 in Y291A-containing cells. Overall, these results demonstrate that pY291 and the aromatic side chain at this position are dispensable for cell death signaling and that the net charge at this site is more important for the protein's function than the detail of the side chain structure.

### Death Domain Phosphorylation Confers Inter- and Intramolecular-Dominant Anti-Apoptotic Activity to Fas

Having established that Y291D mutation emulated the negative charge of pY291 independently of the side chain size reduction and loss of aromatic ring, we used the mutant as a proxy to examine how adding negative charge to this site by phosphorylation could modulate Fas signaling.

**Fig 3:**
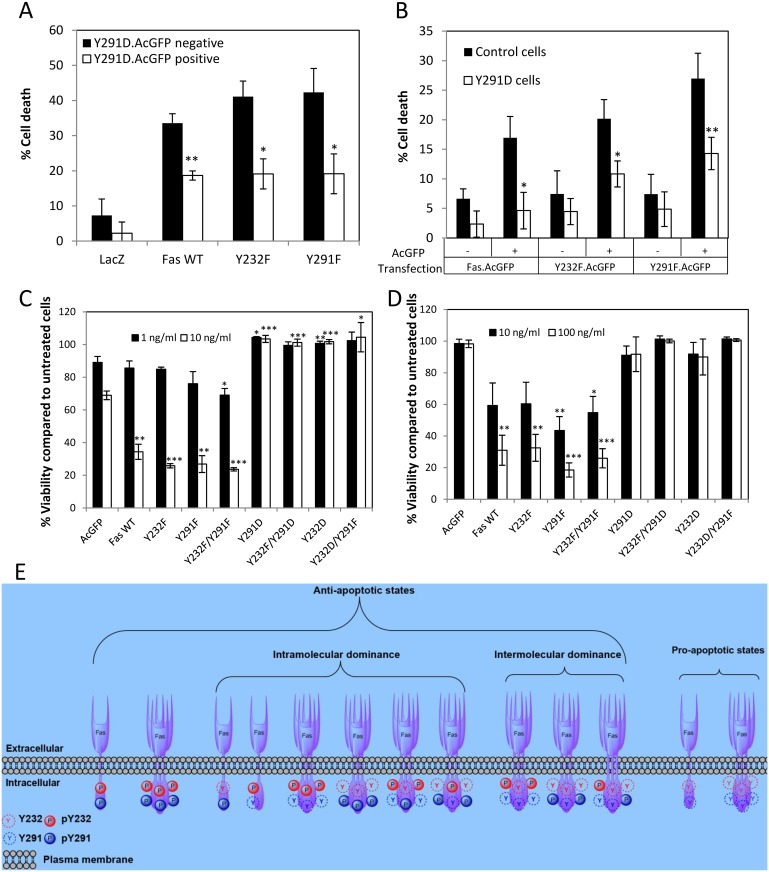
Introduction of negative charge to the 291 position results in a dominant inter- and intra-molecular anti-apoptotic capacity to Fas. (A) SW480 cells stably expressing V5-tagged LacZ and “dead-on” Fas proteins (wild-type, Y232F, or Y291F) were transiently transfected with AcGFP-tagged Y291D Fas for 24 h and subsequently treated with 8 ng/ml FasL+M2 or left untreated for 4 h. Cells were then stained with propidium iodide (PI) and analyzed by flow cytometry for cell death based on membrane permeability. The percentages of PI-positive cells (dead cells) due to FasL treatment in the Y291D.AcGFP-positive and Y291D.AcGFP-negative cell populations are compared after subtracting the percentage of spontaneous cell death in untreated cells. Note an increased resistance to FasL-induced cell death when Y291D.AcGFP Fas was introduced in cells that stably expressed “dead-on” Fas. Values represent means ± SEM from three independent experiments (* *p* < 0.05, ** *p* < 0.01, unpaired *t* test, compared to Y291D.AcGFP negative cells). (B) SW480 cells stably expressing V5-tagged LacZ (control) and “dead-off” Fas Y291D were transiently transfected with AcGFP-tagged “dead-on” Fas (wild-type, Y232F, or Y291F) for 24 h and subsequently treated with 8 ng/ml FasL+M2 or left untreated for 4 h. Cells were then stained with PI and analyzed for cell death by flow cytometry. The percentages of PI-positive cells in the “dead-on” Fas.AcGFP-negative and -positive cell populations are compared after subtracting the percentage of spontaneous cell death in untreated cells. Values represent means ± SEM from three independent experiments (* *p* < 0.05, ** *p* < 0.01, paired *t* test, compared to control cells). Note that compared to control cells, cells stably expressing “dead-off” Y291D Fas were more resistant to the increase in FasL-induced cell death brought about by the introduction of “dead-on” Fas.AcGFP species. (C) SW480 and (D) SW620 cells stably expressing AcGFP or AcGFP-tagged wild-type or indicated mutant Fas proteins were left untreated or treated with indicated concentration of FasL crosslinked with M2 for 24 h and subjected to viability measurement by WST-1 assay. Cell viability is presented as percentage compared to untreated control cells. The presence of Y—>D mutations completely inhibited FasL-induced cell death. Values represent means ± SEM from three independent experiments (* *p* < 0.05, ** *p* < 0.01, *** *p* < 0.001, unpaired *t* test). (E) A diagram depicting different states of Fas, with respect to its ability to transmit an apoptotic signal, as affected by its death domain phosphorylation. Examples of possible dominant-negative scenarios are given. Numerical values underlying the data summary displayed in this figure can be found in S1 Data.

To further demonstrate the anti-apoptotic effect exerted by the negatively charged 291 residue in the Fas signaling, we introduced Y291D mutant Fas (death-off) into cells that stably expressed wild-type, Y232F, or Y291F mutant Fas (death-on) whose apoptotic capacity had been established (Fig 2). We found that the expression of Y291D Fas exhibited a clear dominant-negative effect on all “death-on” Fas species investigated, reducing the level of dead cells by approximately 50% (Fig 3A). In line with these data, when we introduced the “death-on” Fas species to cells stably expressing Y291D, a clear reduction in apoptosis-inducing capacity of the “death-on” Fas was observed when compared to cells expressing the control vector (Fig 3B). The data suggest that a subset of Fas that is phosphorylated can extend its inhibitory effect to an unphosphorylated Fas population, rendering it inefficient in inducing apoptosis.

Since both Y232 and Y291 can be phosphorylated, we examined whether phosphorylation of both tyrosines is required to turn off the death signal. Double mutations of Y232 and Y291 in SW480 and SW620 cells (Fig 3C and 3D) showed that negative-charge mutation at the 291 position alone was sufficient to completely block Fas-mediated cell death. The cell death inhibition in Y232F/Y291D mutant cells shows that maintaining unphosphorylated Y232 could not override the cell death blockage by Y291D mutation. This implies that FasL-induced apoptosis is rendered possible only when Y291 is dephosphorylated and that pY291 exerts dominant-negative effect on this apoptotic process.

While it was evident that dephosphorylation at both Y232 and Y291 (Y232F/Y291F) allowed efficient FasL-induced apoptosis (Fig 3C and 3D), it was unclear whether double tyrosine dephosphorylation was essential for this signal or if single dephosphorylation at Y291 sufficed for the apoptotic signal to proceed. While Y232D mutation partially presented the anti-apoptotic effect of side chain size reduction, the additional anti-apoptotic effect of negative charge at 232 site could still be observed (Fig 2A and 2B). Thus, the observation that FasL-induced cell death in cells carrying Y232D/Y291F mutants remained completely blocked (Fig 3C and 3D) points toward the likelihood that pY232 also exerts dominant-negative effect on FasL-induced apoptosis. That the single dephosphorylation at 291 residue could not override the anti-apoptotic effect of negative-charge addition at 232 residue suggests that double dephosphorylation at both Y232 and Y291 is required for FasL-induced apoptosis. Fig 3E depicts some dominant-negative scenarios in which: 1. an intramolecular dominant-negative effect on apoptosis occurs in a Fas molecule when at least one of the death domain tyrosines is phosphorylated; and 2. an intermolecular dominant-negative effect occurs when Fas molecules carrying at least one death domain pY dominant-negatively suppress the apoptotic function of Fas molecules in the pro-apoptotic state (i.e., having both death domain tyrosines dephosphorylated).

### Y291 Phosphorylation Primes Clathrin-Mediated Endocytosis of Fas

**Fig 4:**
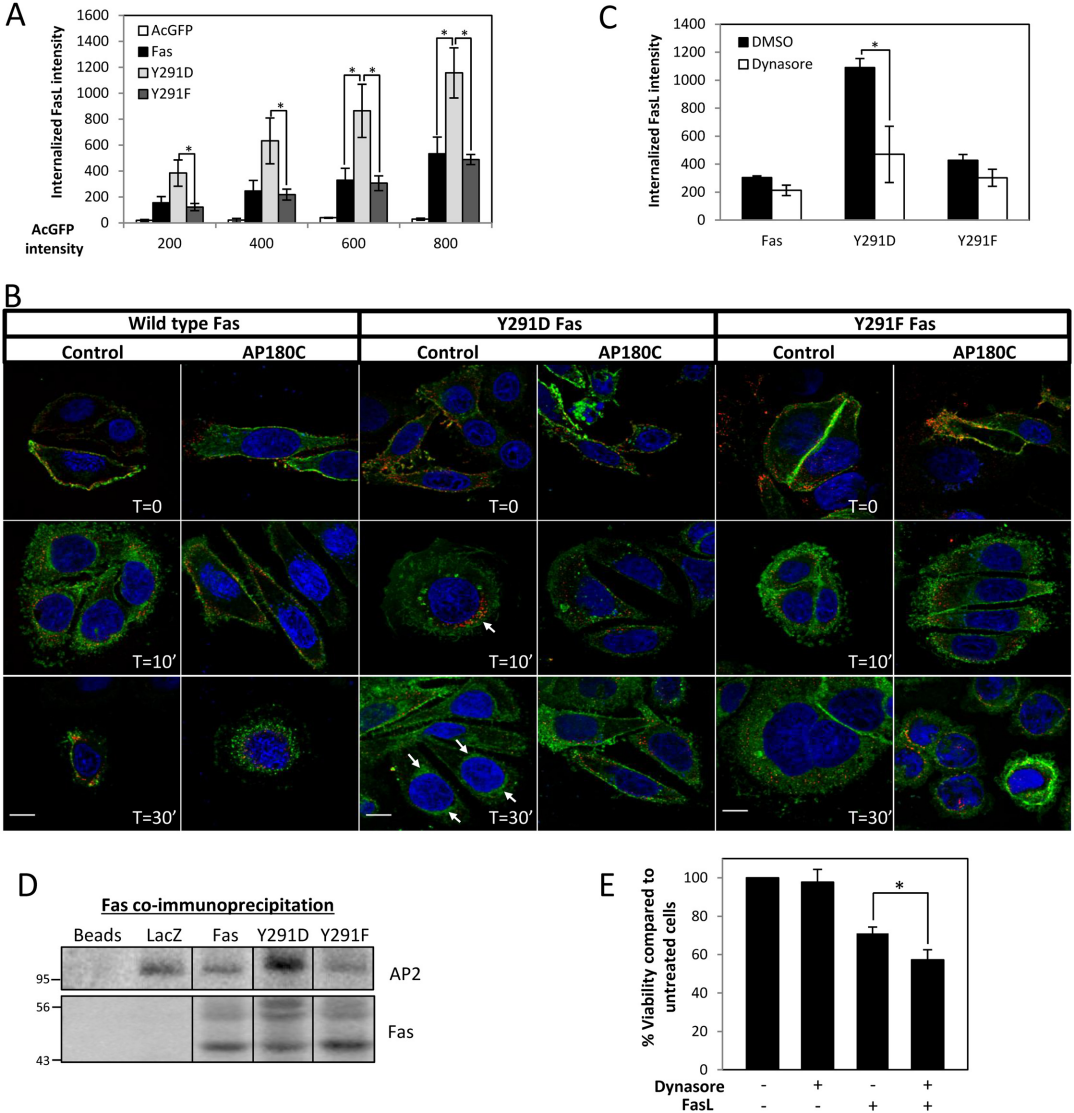
The negative charge of phosphorylated Y291 can promote clathrin-dependent endocytosis (CDE) of Fas upon FasL engagement. (A) Flow cytometry analysis of internalized FasL in SW480 cells overexpressing AcGFP or AcGFP-tagged Fas proteins (as indicated). The extent of FasL internalization was evaluated in cells that expressed equivalent levels of Fas by gating based on the expression level of AcGFP. Results are presented as intensity of internalized FasL in indicated cell lines at different expression levels of AcGFP-tagged Fas or control AcGFP. Note an increase in FasL internalization in SW480 cells overexpressing Y291D.AcGFP Fas. Means ± SEM from three independent experiments are shown (* *p* < 0.05, unpaired *t* test). (B) SW480 cells overexpressing indicated AcGFP-tagged Fas proteins were transiently transfected with AP180-C or control vector before subjected to the internalization assay and imaged by a spinning disk confocal microscope. Color-combined images are presented (green, Fas.AcGFP; red, crosslinked FasL; blue nucleus, scale bar = 10 μm, arrows indicate perinuclear region). Unmerged images showing each channel separately are provided in S5 Fig. (C) SW480 cells expressing indicated AcGFP-tagged Fas proteins were pretreated with vehicle (DMSO) or 100 μM dynasore 30 min before FasL internalization assay and flow cytometry analysis. Fluorescence intensity of internalized FasL of cells with equivalent AcGFP or AcGFP-tagged Fas expression is presented (AcGFP intensity ~600). Means ± SEM are shown (* *p* < 0.05, paired *t* test). (D) Postnuclear supernatants from SW480 cells expressing V5-tagged LacZ or Fas proteins were subjected to coimmunoprecipitation with anti-Fas antibody, SDS-PAGE, and immunoblotting by indicated antibodies. Note that the low level of immunoprecipitated Fas from the LacZ sample could be visualized on the image from longer exposure. (E) SW480 cells expressing V5-tagged wild-type Fas protein pretreated with vehicle (DMSO) or 10 μM dynasore for 30 min were incubated with 10 ng/ml FasL crosslinked with M2 for 24 h before cell viability test by WST-1 assay. Percentage of viability compared to untreated control cells is shown. Means ± SEM from three independent experiments are presented (* *p* < 0.05, unpaired *t* test). Numerical values underlying the data summary displayed in this figure can be found in S1 Data.

The ^291^YDTL motif of Fas has been suggested as a putative tyrosine (Y)-based sorting motif (Yxxϕ; ϕ, a bulky amino acid; x, any amino acid) for clathrin-dependent endocytosis (CDE) [19]. The Y in the motif is essential for binding to μ2 subunit of AP-2 and, in most cases, cannot be substituted by other aromatic acid residues or pY (review [20]). Our data demonstrated that neither Y291D nor Y291F mutation impaired FasL internalization (Fig 4A). Moreover, Y291D Fas expression resulted in a more efficient FasL uptake, indicating that the added negative charge favored the process and, thus, raising doubt regarding the function of Y291 as a critical component of a Y-based sorting motif for CDE. To address these issues, we investigated the involvement of CDE in FasL/Fas uptake. Our synchronized internalization study by immunofluorescence confirmed that neither Y291D nor Y291F inhibited FasL uptake upon its engagement and that the uptake was more efficient with Y291D mutant (Fig 4B). The rapid FasL uptake (within 10 minutes of activation) was accompanied by the transport of a population of Fas to the perinuclear region (Fig 4B). In cells carrying wild type and Y291F Fas, the inhibition of CDE by overexpression of a truncated form of AP180 protein (AP180-C), which blocks the recruitment of clathrin to the plasma membrane [21], caused only a small delay in FasL uptake, which was completed by 30 min of activation. This indicated that the inhibition of Fas/FasL uptake by CDE could be compensated by an alternative pathway, as we previously reported [22]. In contrast, AP180-C expression in cells expressing Y291D Fas strongly inhibited the FasL/Fas complex uptake along with the transport of Y291D Fas to the perinuclear region. Similarly, disrupting dynamin-dependent endocytosis by dynasore, a potent inhibitor to dynamin GTPase activity, led to a strong reduction of FasL/Fas uptake in cells carrying Y291D Fas but not in those carrying wild-type or Y291F Fas (Fig 4C). These data demonstrate that FasL uptake and perinuclear transport of Y291D Fas relied on dynamin-dependent CDE. Unlike in the case of wild-type and Y291F Fas, in which dynamin-independent, clathrin-independent endocytosis (CIE) could compensate for the CDE blockage, the negatively charged Y291D committed the internalization of Fas to CDE. This implies that the constitutive pY291 engages Fas trafficking to CDE exclusively, hence the loss of the flexibility to carryout FasL-activated trafficking via compensatory CIE processes.

Our observation that Y291D even promoted CDE in these cells suggests that Y291 may participate in CDE as a part of another sorting motif. We analyzed amino acids flanking Y291 and found the similarity between the acidic dileucine (LL) sorting motif (D/E)xxxL(L/I) and Fas sequence ^289^EAYDTLI^295^. It is possible that the negative charge of Y291D mutation promoted the interaction between Fas and CDE adaptor proteins that binds the LL motif, such as AP2. Coimmunoprecipitation showed that overexpression of Y291D Fas, but not wild-type or Y291F Fas, increased the association of Fas with AP2 (Fig 4D), suggesting that the negative charge of pY291 may promote the sorting function of the LL motif. This is in line with the importance of phosphorylation in the LL motif in the internalization process, which has been previously reported [23–27]. That inhibiting dynamin-dependent CDE with dynasore sensitized cells to FasL-induced cell death (Fig 4E) also suggests that the function of pY291 in dynamin-dependent CDE contributed to the pro-survival signal of Fas.

### Death Domain Tyrosine Phosphorylation Is Vital to FasL-Induced Non-Death Signaling of Fas

**Fig 5:**
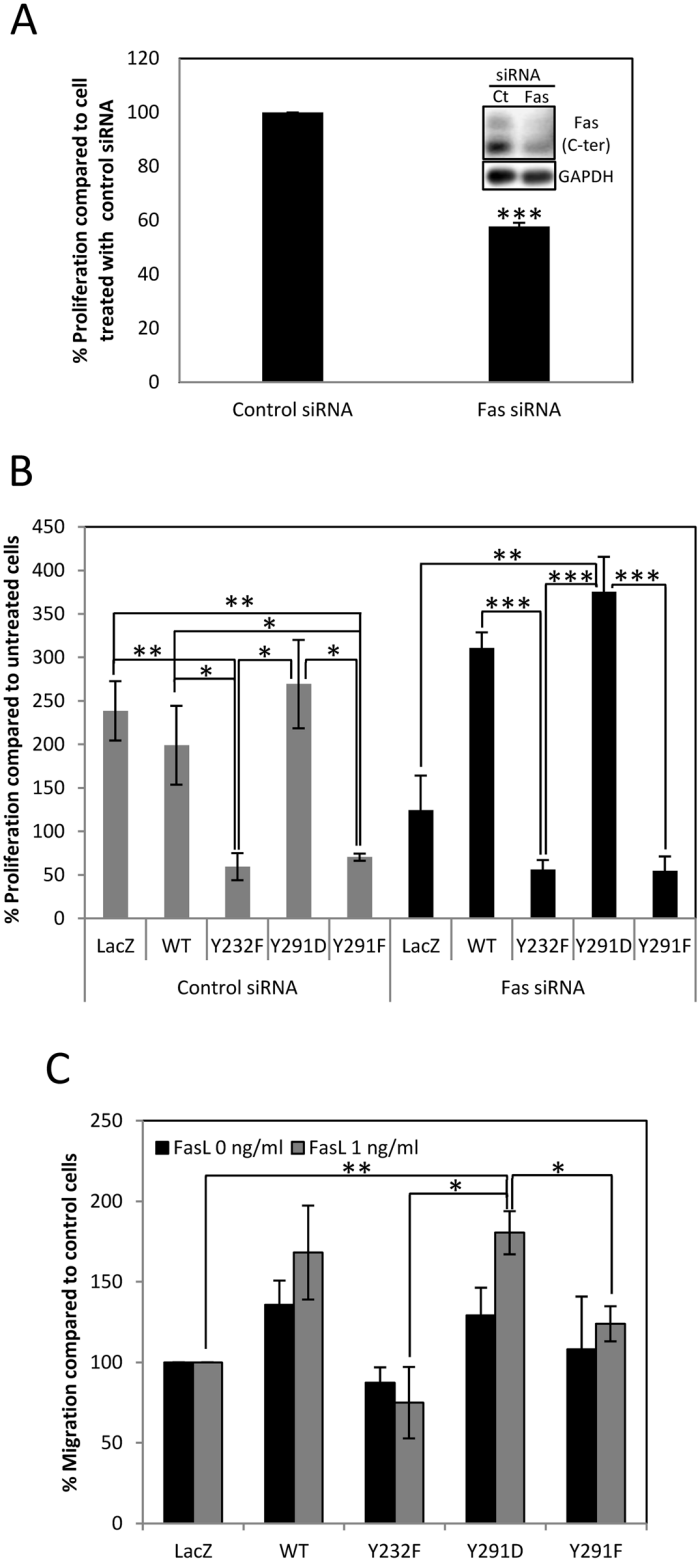
Fas death domain pY is important for non-death functions of Fas. (A) SW480 cells were transiently transfected with control or Fas siRNA before BrdU incorporation analysis by using microplate-based method. Means ± SEM of three independent experiments are shown (*** *p* < 0.001, unpaired *t* test). Typical reduction of Fas level, based on immunoblot, is shown in the inset. (B) SW480 cells stably expressing control (LacZ) or indicated Fas proteins carrying silent mutations at the site targeted by an siRNA against Fas were transiently transfected with control or Fas siRNA for 48 h. They were subsequently synchronized to G1 phase by serum deprivation for 24 h and then treated with 1 ng/ml of sFasL for 30 min before analysing the increase in proliferation by BrdU incorporation measurement using microplate-based method. Means ± SEM of three independent experiments are shown (* *p* < 0.05, ** *p* < 0.01, *** *p* < 0.001, unpaired *t* test). (C) Fluorescently prelabeled SW480 cells were subjected to Boyden chamber migration assay using FluoroBlok membrane inserts. Cells were untreated or induced to migrate by 1 ng/ml sFasL for 2 h. Cells that migrated through the membrane were fixed, imaged, and counted. Data were presented as percentage of cells that migrated through the membrane compared to control cells (LacZ). Means ± SEM of three independent experiments are shown (* *p* < 0.05, ** *p* < 0.01, unpaired *t* test). Numerical values underlying the data summary displayed in this figure can be found in S1 Data.

The contribution of Fas signaling to colorectal cell proliferation was demonstrated as Fas knockdown by siRNA led to a decrease in BrdU incorporation (Fig 5A) and sublethal doses of FasL increased viability of the cells (S6A Fig). To determine the role of Y232 and Y291 phosphorylation in FasL-induced proliferation by site-directed mutagenesis while minimizing the interference from endogenous Fas in SW480 cells, we used stable SW480 cell lines overexpressing Fas proteins that carried silent mutations in the region targeted by an siRNA against Fas. This was to allow the reduction of background signals from endogenous Fas while maintaining that of overexpressed Fas. In cells treated by control siRNA, the FasL-induced proliferation depended on Fas pY, since abolition of tyrosine phosphorylation by the expression of Y232F and Y291F Fas reduced BrdU incorporation, while mimicking the negative charge of pY by Y291D Fas expression did not (Fig 5B). The specific effects of Fas pY mutations in proliferation were confirmed in cells treated with Fas siRNA to reduce background proliferative signals from endogenous Fas. Following Fas siRNA treatment, the FasL-induced proliferation was reduced in control cells, while it increased significantly in cells carrying Y291D mutation, suggesting an active role of pY291 in a proliferative signal of Fas. As found for cells not treated with Fas siRNA, FasL-induced proliferation decreased in cell carrying Y232F and Y291F mutation that were subjected to Fas siRNA treatment, demonstrating a strong inhibitory role of Fas Y232 and Y291 dephosphorylation in FasL-induced proliferation. The importance of pY291 in FasL-induced proliferation was also supported by our observation that the expression of Y291D Fas led to an increase in viability when cells were treated with sublethal doses of FasL, while the expression of Y291F produced the opposite effect (S6B and S6C Fig).

In addition to promoting FasL-induced proliferation, phosphorylation of death domain tyrosine also promoted FasL-induced cell migration. Using Boyden chamber migration assay, we found that cells that overexpressed Y291D Fas mutant exhibited increased migration ability induced by FasL when compared to control cells and cells that expressed unphosphorylable mutants (Fig 5C).

### Phosphorylation of Y232 and Y291 Is Regulated by Src Family Kinases (SFKs) and Protein Tyrosine Phosphatase SHP-1

**Fig 6:**
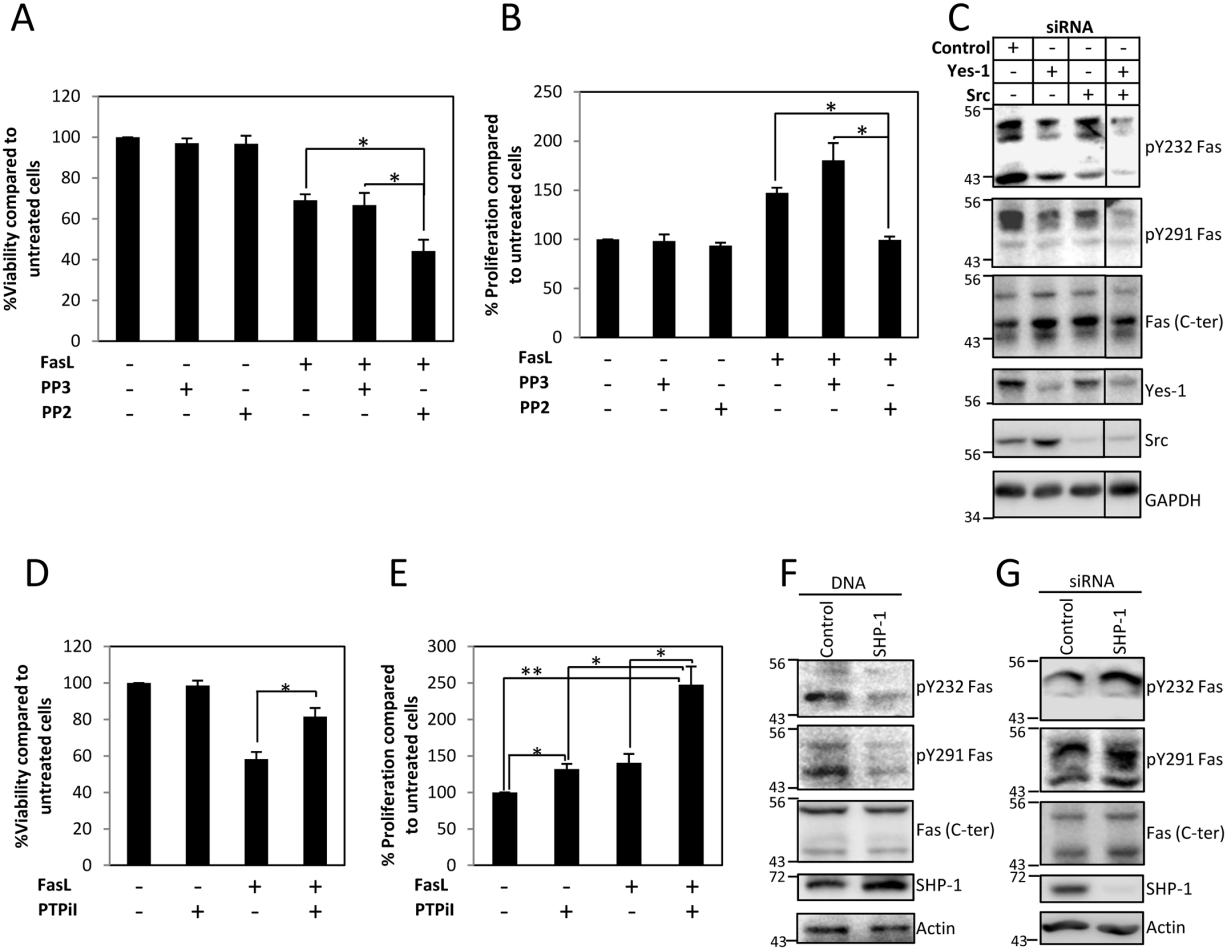
Fas death domain tyrosine phosphorylation is regulated by Src family kinases (SFKs), Src and Yes-1, and protein tyrosine phosphatase SHP-1. (A) SW480 cells expressing V5-tagged Fas protein were pretreated with vehicle (DMSO), 10 μM PP3 (negative control for PP2), or 10 μM PP2 for 30 min and then incubated with 10 ng/ml FasL crosslinked with M2 for 24 h before subjected to cell viability analysis using WST-1 assay. Percentages of viability compared to untreated control cells are shown as means ± SEM from three independent experiments (* *p* < 0.05, unpaired *t* test). (B) SW480 cells were synchronized to G1 phase by serum deprivation for 24 h and then treated with vehicle (DMSO), 10 μM PP3 (negative control for PP2), or 10 μM PP2 for 30 min, followed by 0.1 ng/ml of sFasL for 4 h before analyzing the proliferation by BrdU incorporation measurement using microplate-based method. Means ± SEM of three independent experiments are shown (* *p* < 0.05, unpaired *t* test). (C) SW480 cells were transiently transfected with control siRNA or siRNA against Src, Yes-1, or Src and Yes-1 for 72 h. Cell lysates were then collected and subjected to SDS-PAGE and immunoblotting with indicated antibodies. (D) SW480 cells expressing V5-tagged Fas protein were pretreated with vehicle (DMSO) or 50 μM PTPiI for 30 min and then incubated with 10 ng/ml FasL crosslinked with M2 for 24 h before being subjected to cell viability analysis using WST-1 assay. Percentage of viability compared to untreated control cells are shown as means ± SEM from three independent experiments (* *p* < 0.05, unpaired *t* test). (E) SW480 cells were synchronized to G1 phase by serum deprivation for 24 h and then treated with vehicle (DMSO) or 50 μM PTPiI for 30 min followed by 0.1 ng/ml of sFasL for 1 h before analyzing the proliferation by BrdU incorporation measurement using microplate-based method. Means ± SEM of three independent experiments are shown (* *p* < 0.05, ** *p* < 0.01, unpaired *t* test). (F) SW480 cells were transfected with control vector or SHP-1 for 24 h and (G) with control siRNA or siRNA against SHP-1 for 48 h, then synchronized in G1 phase by serum deprivation for 24 h before treatment with 0.1 ng/ml FasL for 5 min. Cell lysates were then collected and subjected to SDS-PAGE and immunoblotting with indicated antibodies. The specificity of anti-pY232 and anti-pY291 Fas antibodies is demonstrated in S11 Fig. Numerical values underlying the data summary displayed in this figure can be found in S1 Data.

Based on previously suggested involvement of SFKs in Fas signaling [11,28–29], we examined their influence on FasL-induced cell death. Inhibiting SFKs by a Src family kinase inhibitor, PP2, sensitized cells to FasL-induced cell death (Fig 6A). On the other hand, PP2 also significantly reduces FasL-induced proliferation (Fig 6B). The potentiation of FasL-induced cell death and the inhibition of FasL-induced proliferation by PP2 implied the role of SFKs in the phosphorylation of Fas death domain tyrosines. To further identify the SFKs involved, we subjected the cells to siRNA against Src and Yes-1 and found that suppressing either Src or Yes-1 could somewhat reduce the levels of pY232 and pY291 Fas (Fig 6C). However, the effect was more pronounced when both Src and Yes-1 were simultaneously suppressed. This reflects the redundancy of SFK activities in the Fas tyrosine phosphorylation process, since suppression of Src or Yes-1 alone was not as efficient as suppressing both kinases in reducing pY232 and pY291 Fas. The functional redundancy among SFKs is well recognized [30], and this notion is also supported by our observation that overexpression of either Src or Yes-1 could increase the level of pY232 and pY291 Fas (S12 Fig).

The protein tyrosine phosphatase, SHP-1, has been implicated in Fas signaling [31]. Therefore, we investigated its involvement in Fas tyrosine phosphorylation process. We found that inhibiting SHP-1 activity by protein tyrosine phosphatase inhibitor I (PTPiI) protected the cells from FasL-induced cell death (Fig 6D) while promoting FasL-induced proliferation (Fig 6E). This implied that SHP-1 might negatively regulate the phosphorylation of Fas death domain tyrosines. We further confirmed the role of SHP-1 in Fas dephosphorylation by demonstrating that overexpressing SHP-1 protein decreased pY232 and pY291 levels (Fig 6F) while suppressing SHP-1 expression by siRNA resulted in the opposite effect (Fig 6G).

### Fas Tyrosine Phosphorylation Profiles Can Correlate to Different Contexts of Human Cancers

**Fig 7:**
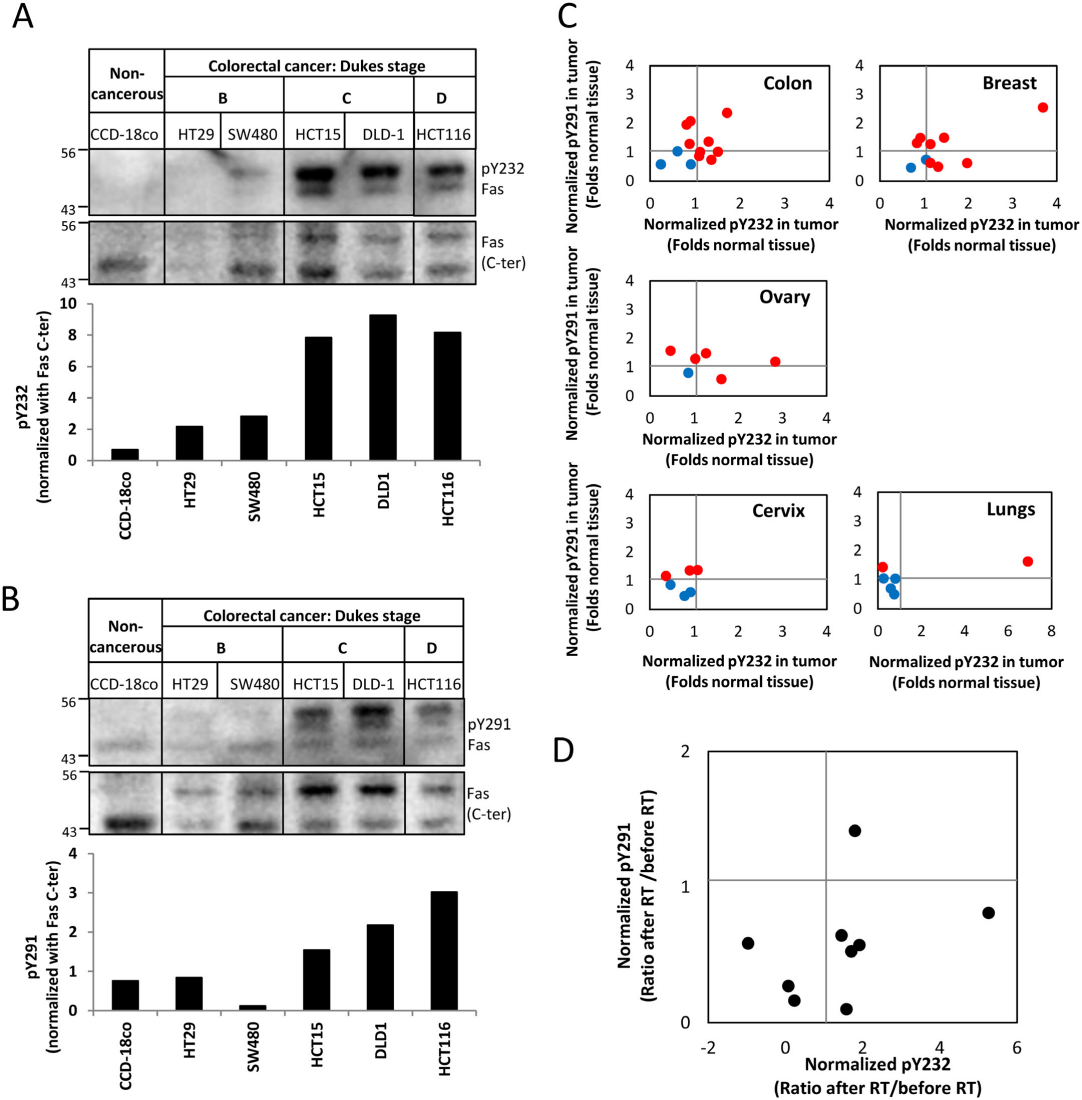
Fas tyrosine phosphorylation profiles in human cancers is context-dependent. (A) and (B) Lysates prepared from human colon cells, noncancerous and cancerous, were subjected to SDS-PAGE and immunoblotting with indicated antibodies. The band intensity of the pY232, pY291, and C-terminal (C-ter) of Fas (between 43 and 56 kDa) was quantified using ImageStudio software. The values of pY232 and pY291 intensity after normalization with Fas (C-ter) intensity are presented as normalized pY232 and pY291 (lower panels). (C) Matched cancer and normal tissue lysates, each pair from the same individual, were subjected to SDS-PAGE and immunoblotting with antibodies against pY232, pY291, and C-terminal (C-ter) of Fas. The pY232 and pY291 levels were measured by densitometric analysis and normalized as in (A) and (B). The ratio between normalized pY232 or pY291 Fas from cancer tissue and normal tissue was calculated for each patient, and values were plotted as folds normalized pY232 and pY291 from normal tissue (i.e., normalized values of pY232 and pY291 from normal tissues are assigned as 1). Red represents samples having the normalized pY232 and/or pY291 values in cancer tissues ≥ 1.1 folds of normal tissues (of which enhanced pY232 and/or pY291 level may be indicative of dominant survival mode of Fas signaling); blue represents samples having both normalized pY232 and pY291 values < 1.1 (of which pY232 and pY291 levels less than or equal to normal may be indicative of the apoptosis-ready mode of Fas signaling). (D) Lysates of rectal cancer tissues before and after radiotherapy (RT) from each of the nine patients were subjected to SDS-PAGE and immunoblotting with antibodies against pY232, pY291, and C-terminal (C-ter) of Fas. The normalized values of pY232 and pY291 were calculated as in (A) and (B). Each value presented is the ratio between normalized pY-Fas after RT and before RT of each patient (i.e., normalized values of pY232 and pY291 of tumors obtained before RT are assigned as 1). Numerical values underlying the data summary displayed in this figure can be found in S1 Data.

By comparing several colon cell lines, we found that the relative Fas pY levels tended to increase with the cancer progression (Fig 7A and 7B), implying that Fas pY might correlate to some contexts of human cancers. We therefore examined the relative levels of pY232 and pY291 in malignant tissues when compared to corresponding normal tissues of patients diagnosed with different types of cancers. We found that most patients having cancer of the colon, breast, or ovary also had an increased level of pY232 and/or pY291. However, this was not the case in patients having cancer of the cervix or lungs (Fig 7C). These diverse Fas pY profiles in different cancer types suggests that Fas signaling modes may be cancer type-dependent. Additional evidence supporting the involvement of Fas pY in human cancer comes from our observation that pY291 Fas levels decreased while pY232 Fas levels increased in the majority of rectal tumors after radiotherapy (± concurrent chemotherapy, Fig 7D), suggesting distinct regulation and functions of pY232 and pY291 in Fas signaling in rectal cancer in response to cancer therapy.

## Discussion

### Fas Death Domain Tyrosine Phosphorylation and Outcome of Fas Signaling

The functions of different phospho-tyrosines of Fas have not been distinguished to date. Using comparative genomics to guide functional analysis of Fas pY, in conjunction with site-specific detection of the phosphorylated death domain tyrosines, Y232 and Y291, we show that the phosphorylation of both death domain tyrosines in human Fas is dispensable for FasL-induced apoptosis. Our findings in human colorectal cells and B-cells that demonstrate this claim are well supported by evolution data and functional data from other animal cell models, including macaques [10] and rats [8,11]. Guided by comparative genomics, which reveals an unconventional cysteine substitution for Y291 in primates and rodents, we show that the net charge at this site is more important for the protein's function than the size or details of the amino acid side chain. This has created the possibility of using acidic amino acid substitution as a proxy for pY291 and of demonstrating that pY291 is, rather, a pro-survival mechanism that confers apoptosis resistance and proliferative advantage while its dephosphorylation permits apoptotic process.

The substitution of C for Y at 291 residue in old world monkeys is unique among the three Fas tyrosines found in primates. It suggests a low structural requirement from this tyrosine and that its role is, rather, in functional specificity. This amino acid exchange in Fas orthologs in closely related species may appear surprising and drastic considering amino acid sizes and properties. However, it serves as an example that, for certain protein functions, Y can be exchanged for a small, non-aromatic amino acid and that such Y (or pY) may have evolved from a smaller amino acid, as previously demonstrated [12]. Our finding that small amino acid substitutions occur at pY sites of Caspase 8 and are common in FAP-1 (S7 and S8 Figs) supports this point of view. The shift from small amino acids to (p)Y of Fas and other proteins in the Fas signaling pathway in primates implies a preferential shift to pY switch systems that can confer their functional plasticity and specificity in these species.

### Functions of Fas Death Domain Tyrosines

We show that pY232 and pY291 of Fas have common features. They (1) are dispensable for Fas-induced apoptosis and (2) dominant-negatively inhibit apoptosis. Using the proxy Y291D, we also show that pY291 can promote FasL-induced cell proliferation and, thus, present pY291 as a reversible anti-apoptotic/pro-survival switch of Fas. This switch mechanism involves the function of pY291 in preventing FasL-induced DISC formation (Fig 2E) and promoting CDE of Fas (Fig 4).

Previous work showed that the Y291F mutation of human Fas (hFas) in murine cells inhibited the downregulation of the hFas-antibody complex [19]. Since the ^291^YDTL motif is consistent with the Y-based sorting motif for CDE, one may infer that this decrease in the antibody-induced Fas downregulation was caused by Y → F substitution in the motif. However, we found that, in human cells, Y291F mutation did not inhibit the FasL uptake (Fig 4). Likewise, Y283F mutation of murine Fas (corresponding to Y291F in hFas) in murine T cells did not inhibit the downregulation of the surface Fas-FasL complex (S9 Fig). Additionally, using the Y291D mutation, we further provide evidence suggesting that pY291 could enhance the uptake of the receptor via AP2-mediated CDE, which was important to its anti-apoptotic role. The fact that substitution of Y by other amino acids allowed Fas/FasL uptake by CDE suggests that the FasL-induced Fas uptake did not depend on Y291 as a part of the Y-based sorting motif but possibly of other motifs such as the acidic LL motif. Our finding is in line with previous reports for Vpu protein from HIV-1 subtype C [32], in which the tyrosine was dispensable for the protein's cell surface transport but important for viral replication, while the LL motif was crucial for cell surface transport.

Additionally, we offer an insight into distinct functions of Y232 and Y291 and their respective phosphorylation, which has not been addressed thus far: (1) the aromatic side chain of Y232 may contribute more to the structural integrity of the protein than that of Y291 (Fig 1A); (2) the size and details of the hydroxyl aromatic side chain of Y232 are essential for the functions of Fas, while the charge of Y291 is more important than the size and details of the aromatic side chain (Fig 2); and (3) pY232 and pY291 are distinctly regulated in different types of cancer (see below).

### Regulation of Death Domain Tyrosine Phosphorylation of Fas by SFKs and SHP-1

SFKs are important mediators of tumor cell proliferation and survival and are involved in Fas signaling (Fig 6A, [33–34]). Yet, how activities of SFKs exert an effect on Fas has been unclear thus far. We reveal an intricate regulation of Fas death domain phosphorylation by SFKs, leading to the inhibition of the apoptotic signal of Fas, and, thus, provide the first mechanistic link between SFKs, major drivers of tumor development and progression, and the control of Fas multisignaling. This is of clinical significance because, in tumors from glioblastoma multiforme patients, the expression of Yes and phosphorylation of SFKs, as well as an enhanced FasL expression, were observed in the zone of tumor–host interaction, suggesting their roles in glioma invasion [34]. This is in concert with the concept presented herein that, in cancer, Fas-mediated survival signaling is promoted by SFK-dependent tyrosine phosphorylation.

Concerning the Fas pY dephosphorylation, it has been proposed that SHP-1 might be involved in this process in neutrophil, since it is associated with wild-type human Fas but not Y291A mutant in mouse lymphoblastic cells, and human SHP-1 from several cell lines could associate with phosphorylated peptides corresponding to the YxxL motif of death receptors [7]. We show that, in colorectal cells, pY232 and pY291 dephosphorylation is mediated by SHP-1, which has been shown to effectively dephosphorylate Src substrates [35] and to negatively regulate colonic cells proliferation [36]. Our data support the notion that the pY-based proliferative/apoptotic switch system of Fas is regulated via phosphorylation by SFKs and dephosphorylation by SHP-1, similar to that of caspase-8 [37–38]. This emphasizes the importance of the SFKs/SHP-1-based phosphorylation/dephosphorylation mechanism in the Fas multisignaling pathway.

### Regulation and Functions of Death Domain Tyrosines and Their Implications in Cancer

Multimodal signaling of Fas has been demonstrated in many cancer cell types, including colon [39], breast [40], and glioblastoma [34]. Currently, both pro-apoptotic and pro-survival roles of Fas are bases of therapeutics that aim either to activate Fas signaling (APO010 agent targeting extracellular domain of Fas) [41] or to inhibit Fas signaling triggered by FasL (APG101 targeting FasL) [42]. These approaches face a major challenge, since it has been unclear what determines the outcome of Fas signaling and when one role of Fas will dominate the other. Our observation that colon, breast, and ovarian malignant tissues from most patients we tested had higher levels of pY232 and/or pY291 than their corresponding normal tissues suggests the probability that the pro-survival signal of Fas may dominantly operate in these cancers. On the other hand, the opposite observation for tumors from lung cancer patients suggests the probability that Fas may not contribute to the pro-survival signal in lung cancer (Fig 7C). Furthermore, we provide evidence that pY232 and pY291 can be distinctly regulated in cancer by showing opposing changes in their levels in rectal tumors in response to radiotherapy (RT ± chemotherapy). The reduction of pY291 following the treatment may suggest a decrease in the FasL-induced pro-proliferative/anti-apoptotic signal of Fas conferred by pY291, whereas the increase in pY232 may suggest an increase in other signaling events that involve the function of pY232. This may include the involvement of pY232 in the cell cycle phase, since we also observed a Fas-dependent G2/M accumulation that depended on the phenolic hydroxyl group of Y232 (Figs 2A and S10). This G2/M accumulation was associated neither with the resistance to FasL-induced apoptosis (Fig 2) nor an increase in FasL-induced cell proliferation (Fig 5B), suggesting an additional role of pY232 that is independent of FasL-induced apoptotic and proliferative functions of the protein.

Data presented here were obtained from a small number of patients. Thus, generalizations about the various extents of Fas phosphorylation in different types of cancer should be made with caution. However, our data revealing that the outcome of Fas signaling is determined by its pY status of the death domain and that the Fas pY status may differ among different cancer types and may respond to anticancer treatment provide a basis for further studies in larger sets of human cancer samples, as well as an opportunity to develop a practical means to predict the outcome of Fas signaling in different pathologies that can lead to the use of Fas pY screening to aid Fas-related therapeutic design and maximize the chance of therapeutic success.

Overall, we provide the delineation of the pY-based control of Fas signaling, revealing differential evolutional criteria of the two death domain tyrosines, their regulatory elements, mechanistic links between this molecular switch system and the cellular outcome, and the implications in cancer. This information has far-reaching consequences, not only in cancer contexts but also in other pathologies in which Fas signaling is involved.

## Materials and Methods

### Ethic Statement

The rectal tumors were obtained from patients providing informed consent under protocols approved by the Clinic Institutional Review Board of the Département Recherche Clinique Innovation et Statistiques (DRIS)–Centre Antoine LACASSAGNE, Nice, France.

Materials and additional detailed methods are listed in S1 Text.

### Flow Cytometry Analysis of Unsynchronized Fas/FasL Complex Internalization

SW480 cells (2.5 x 10^5^ cells/well) were seeded in a 24-well plate for 24 h. The medium was then replaced with fresh RPMI+0.1% BSA containing 1 μg/ml mouse anti-Flag (M2)+ 1 μg/ml donkey anti-mouse Alexa Fluor 647 with or without 100 ng/ml FasL. The cells were then incubated at 37°C for a specified time to allow FasL-triggered stimulation. Thereafter, the plate was transferred to an iced water basin and the activation was stopped by adding ice-cold PBS. FasL that remained on the cell surface was removed using an ice-cold acid-stripping buffer (50 mM glycine, 100 mM NaCl, pH3). After washing with ice-cold PBS, cells were analyzed for the internalized FasL (crosslinked with mouse anti-Flag antibody and Alexa Fluor 647 anti-mouse antibody) by flow cytometer (LSRFortessa, Becton Dickinson). To assess the degree of FasL internalization, the median fluorescence intensity of the background control cells (treated with anti-Flag and Alexa Fluor 647 without FasL) was subtracted from the median fluorescence intensity of the FasL-treated cells to obtain the absolute fluorescence intensity of the detected internalized FasL in the cells.

### Synchronized Fas/FasL Complex Internalization Analysis by Immunofluorescence Microscopy

SW480 Cells (2.5 x 10^5^ cells/well) were seeded in RPMI+10% FBS on coverslip in 24-well plate for 24 h. Cells were then cooled down to 0°C in a refrigerating chamber for 45 min in RPMI+10% FBS+10 mM Hepes. The medium was then replaced with ice-cold RPMI+0.1% BSA+10 mM Hepes (internalization medium) containing 200 ng/ml FasL+1μg/ml mouse anti-FLAG (M2)+1 μg/ml Alexa Fluor-647 anti-mouse antibody, and cells were incubated at 0°C in a refrigerating chamber for 1 h to allow FasL binding. The medium containing excess FasL was then removed, and internalization medium at 0°C was added to the well. The coverslips containing cells for control condition were kept at 0°C in the refrigerating chamber throughout the experiment, while coverslips containing cells destined for activation were rapidly transferred to another 24-well plate containing 0.25 ml of internalization medium per well (maintained at 37°C in an incubator) to trigger the internalization of Fas/FasL complexes. After incubation at 37°C for indicated times, the plate was rapidly transferred from the incubator to the refrigerating chamber, and 1 ml of PBS at 0°C was added to each well to stop the activation. Cells were then rapidly fixed with ice-cold 4% paraformaldehyde for 20 min on ice and counter-stained with DAPI for nuclear detection before mounting on a slide in the presence of mounting medium (Fluoromount, Sigma-Aldrich). Fluorescence images were taken using a spinning disk confocal microscope (Olympus/Andor/Yokogawa system) with a 100×oil/1.4 numerical aperture objective lens. Images were deconvolved with Huygens software (Scientific Volume Imaging).

### Mobility Shift Detection of Phospho-Proteins

Phosphate affinity SDS-PAGE was performed using 7.5% polyacrylamide gels containing 10 μM acrylamide-pendant Phos-Tag (Wako), according to the manufacturer's instructions. Highly phosphorylated proteins migrate more slowly through the gel than less phosphorylated proteins, allowing protein separation based on phosphorylation states.

## Supporting Information

S1 Data Numerical values underlying the quantitative summary in the main and supplementary figures. Numerical values for Figs 2B, 2D, 3A–3D, 4A, 4C, 4E, 5A–5C, 6A, 6B, 6D, 6E, 7A–7D, S1B, S1C, S6A–S6C, S9 and S10 are presented. The data are shown for each figure panel in a separate worksheet.
(XLSX)

S1 Fig Tyrosine phosphorylation of Fas death domain is dispensable in FasL-induced apoptosis in human colorectal cancer cells. (A) SW480 cells stably expressing control protein (LacZ.V5) or indicated Fas.V5 proteins were treated with 10 ng/ml FasL crosslinked with anti-FLAG (M2) for indicated times, and cells lysates were collected and subjected to SDS-PAGE and immunoblotting with indicated antibodies. Initiation and execution of apoptosis is signified by the cleavage of caspase-8, caspase-3, and PARP. (B) Viability of cells treated with indicated doses of crosslinked FasL for 24 h was measured by WST-1 assay and presented as percentage of cell viability compared to untreated control cells. (C) Cells were pre-incubated with pan-caspase inhibitor, zVAD (10 μM), or DMSO for 30 min before incubating for 24 h with or without 10 ng/ml FasL crosslinked with M2 and assessed for cell viability using WST-1 assay. Note that the Y232F and Y291F mutations exhibit the same ability as wild-type Fas to transduce apoptosis in SW480 cells upon stimulation with crosslinked FasL, as shown by the activation of the caspase cascade and PARP cleavage (A) and cytotoxic assay (B). Similar to wild-type Fas, the cell death in cells carrying Y232F and Y291F mutants could be inhibited by the pan-caspase inhibitor zVAD, confirming the caspase-dependent apoptosis transduced by these unphosphorylated mutants (C). Numerical values underlying the data summary displayed in this figure can be found in S1 Data.
(TIF)

S2 Fig Phosphorylation of death domain tyrosine is dispensable for FasL-induced cell death in human acute amyloid leukemic cells. Left panel: flow cytometry analysis of Fas surface expression of WSU cells stably overexpressing control vector (pCR3), Fas, and unphosphorylated mutant Fas proteins (gray, isotype control; black, anti-Fas antibody). Middle panel: flow cytometry analysis of cell death assessed by the loss of plasma membrane integrity (PI staining without cell fixation) without or with activation with 50 ng/ml FasL crosslinked with 1 μg/ml anti-Flag (M2) for 20 h. Numbers shown are percentages of cells with positive staining for PI, representing dead cells. Right panel: flow cytometry analysis of apoptosis based on DNA fragmentation as assessed by the appearance of subG1 cell population (PI staining after cell fixation) without or with activation with 50 ng/ml FasL crosslinked with 1 μg/ml anti-Flag (M2) for 6 h. Numbers indicate percentage of cells that underwent apoptosis, having subG1 DNA content due to DNA fragmentation. Like in SW480 cells, the Y232F and Y291F mutations exhibited the same ability as wild-type Fas to transduce apoptosis in WSU cells upon stimulation with crosslinked FasL.
(TIF)

S3 Fig Features of amino acids used in the site-directed mutagenesis. (TIF)

S4 Fig Fas expression of stable cell lines used in site-directed mutagenesis studies. Flow cytometry analysis showing equivalent levels of Fas surface expression of SW480 cells stably overexpressing V5-tagged proteins (A) and (B), and SW480 cells stably over-expressing Fas V5-tagged proteins carrying silent mutations at the site targeted by a Fas siRNA (C) (gray, isotype control; black, anti-Fas antibody).
(TIF)

S5 Fig Inhibition of CDE by AP180-C expression prevented the trafficking of Y291D Fas to the perinuclear region after activation with FasL. Supplementing the results presented in Fig 4B, unmerged images of SW480 cells subjected to synchronized FasL internalization assay and imaged by spinning disk confocal microscope are shown here. Images from each channel are shown in gray scale. In merged images, green represents AcGFP; red, FasL.
(TIF)

S6 Fig FasL-induced proliferation is promoted by Y291D mutation. (A) Cell viability assay showing that FasL can activate cell proliferation. SW480 cells were synchronized to G1 phase by serum deprivation for 24 h and then left untreated or treated with indicated sublethal doses of soluble FasL (sFasL) for 48 h before viability measurement by WST-1 assay. (B) FasL-induced proliferation was enhanced in cells carrying Y291D Fas mutation. SW480 cells stably expressing V5-tagged LacZ or indicated Fas proteins were synchronized to G1 phase by serum deprivation in RPMI+0.1% BSA for 24 h and left untreated or treated with indicated concentration of uncrosslinked sFasL (ng/ml) for 48 h before viability measurement by WST-1 assay. Data is presented as the percent increase in cell viability after FasL treatment compared to untreated control cells. Values represent means ± SEM from at least three independent experiments (* p < 0.05, *t* test). (C) As in SW480 cells, the expression of Y291D in SW620 cells enhanced the proliferative effect of sFasL, whereas expression of Y291F Fas reduced the proliferative effect of sFasL. SW620 cells stably expressing AcGFP or indicated AcGFP-tagged Fas proteins were synchronized to G1 phase by serum deprivation and then treated with indicated concentration of uncrosslinked sFasL (ng/ml). Cell viability was measured using WST-1 assay. Numerical values underlying the data summary displayed in this figure can be found in S1 Data.
(TIF)

S7 Fig Multiple sequence alignment of caspase-8 proteins from different vertebrates reveals a common cysteine substitution for tyrosine at positions corresponding to tyrosine phosphorylation sites of human caspase-8. Partial alignment of sequences of caspase-8 from different vertebrates is shown. The amino acid positions of tyrosines in human caspase-8 are indicated (.). Only stretches of the sequence encompassing selected tyrosines are shown. The amino acid positions corresponding to tyrosines in human caspase-8 are highlighted. Cysteine and acidic amino acid residues at positions corresponding to the tyrosines of human caspase-8 are boxed in black and red, respectively (*, tyrosine phosphorylation sites: Y293 and Y380).
(TIF)

S8 Fig Multiple sequence alignment of FAP-1 proteins from different vertebrates reveals common amino acid exchanges between cysteine or acidic amino acids and tyrosines in FAP-1. Partial alignment of sequences of FAP-1 from different vertebrates. The amino acid positions of tyrosines in human FAP-1 are indicated (.). Only stretches of the sequence encompassing selected tyrosines are shown. The amino acid positions corresponding to tyrosines in human FAP-1 are highlighted. Cysteine and acidic amino acid residues at positions corresponding to the tyrosines of human FAP-1 are boxed in black and red, respectively.
(TIF)

S9 Fig Y283F mutation in murine Fas did not inhibit Fas/FasL complex uptake in murine T cells. L1210 cells stably expressing wild type or Y283F murine Fas were subjected to synchronized internalization assay as described previously [22,43]. Briefly, crosslinked FasL was allowed to bind to the cell surface at 4°C before the internalization was triggered by raising the temperature to 37°C. The degree of Fas/FasL complex uptake was correlated with the downregulation of Fas/FasL complex from the cell surface by measuring the remaining FasL on the cell surface and normalized by the level of total FasL-bound before internalization was commenced. A lower percentage of FasL remaining on the cell surface indicates higher downregulation (internalization) of Fas/FasL complex. Numerical values underlying the data summary displayed in this figure can be found in S1 Data.
(TIF)

S10 Fig An increase in population of cells in G2/M phase caused by raising Fas expression level requires the phenolic hydroxyl group of Y232. SW480 cells stably overexpressing V5-tagged Fas wild type and Y232 mutant proteins with equivalent Fas surface expression were subjected to cell cycle analysis based on DNA content. Data shown are mean ± SEM values of percent of cells in G2/M phase (DNA level = 4N) from three independent experiments. Numerical values underlying the data summary displayed in this figure can be found in S1 Data.
(TIF)

S11 Fig Specificity validation of antibodies against Fas protein phosphorylated at Y232 and Y291. **(A) Based on Fas siRNA, the antibodies against pY232 and pY291 Fas specifically detect Fas protein.** Separation and detection of phosphorylated Fas protein in lysates from cells transfected with or without Fas siRNA by classical SDS-PAGE and immunoblotting demonstrating that pYFas antibodies detect Fas protein. SW480 cells were transiently transfected with 20 nM of control siRNA or siRNA directed against FAS gene. Cell lysates were then collected after 72 h of transfection and subjected to SDS-PAGE, where protein separation was based on molecular weights, followed by immunoblotting with indicated antibodies. Note a clear reduction in pY232 and pY291 Fas upon Fas silencing. **(B) Based on siRNA and shRNA coupled with phospho-protein mobility shift SDS-PAGE, the antibodies against pY232 and pY291 Fas specifically detect the population of Fas protein that is phosphorylated.** Mobility shift detection of pYFas protein in lysates from cells, with or without Fas Suppression by RNAi, demonstrating that the pYFas antibodies detect pYFas. SW480 cells were transiently transfected with 20 nM of control siRNA or siRNA directed against exon 3 of the *FAS* gene for 72 h (left panel) or stably infected with short hair-pin RNAi (shRNAi) against exon 9 of the *FAS* gene (right panel). Cell lysates were collected and subjected to mobility shift, phosphate affinity SDS-PAGE using acrylamide-pendant Phos-tag^™^, in which proteins were separated based on their degree of phosphorylation, followed by immunoblotting with indicated antibodies. Note that the antibodies against pY232 and pY291 detected slow-migrating Fas bands (upper part of the gel, characteristic of phosphorylated proteins), and the detection was clearly reduced upon the reduction of Fas protein by siRNA or shRNAi, confirming the specificity of the antibodies to phosphorylated Fas protein. **(C) Based on site-directed mutagenesis, the antibodies against pY232 and pY291 Fas detect pY232 and pY291 residues in a site specific manner.** Left panels: The antibody against pY232 Fas detected a significant increase in pYFas levels in the cell lysate obtained from cells overexpressing wild-type Fas, whereas such detection was absent for the lysate from cells overexpressing the unphosphorylable Y232F mutant Fas. Thus, the antibody against pY232 Fas specifically detected Fas protein that was phosphorylated at Y232 residue. Note that cells carrying the Y232F mutant Fas and cells carrying wild-type Fas contained equivalent levels of overexpressed Fas (as seen from an equivalent detection of V5 tag and the C-terminus of Fas). Right panels: Like the antibody against pY232 Fas, the antibody against pY291 Fas detected a significant increase in pYFas level in the cell lysate obtained from cells overexpressing wild-type Fas, whereas such detection was absent for lysate from cells overexpressing the unphosphorylable Y291F mutant Fas. Thus, the antibody against pY291 Fas specifically detected Fas protein that was phosphorylated at Y291 residue. Note that cells carrying the Y291F mutant Fas and cells carrying wild-type Fas contained equivalent levels of overexpressed Fas (as seen from an equivalent detection of V5 tag and the C-terminus of Fas). This validation of site specificity was carried out using SW480 cells stably overexpressing V5-tagged Fas (wild-type or pY mutants) that carried silent mutations at the site targeted by an siRNA against Fas. In order to reduce the background pYFas signal generated by the endogenous Fas, 72 h prior to cell lysate collection, cells were treated with 20 nM of corresponding siRNA against Fas, which reduced the endogenous Fas level while sparing the levels of overexpressed Fas. Cell lysates collected were subjected to mobility shift, phosphate affinity SDS-PAGE using acrylamide-pendant Phos-tag^™^, in which proteins were separated based on their degree of phosphorylation, followed by immunoblotting with indicated antibodies. **(D) Dephosphorylation by calf intestinal phosphatase additionally confirmed that both antibodies against pY232 and pY291 Fas detected phosphorylated proteins.** This is based on the loss of detection when phosphorylated proteins were dephosphorylated by calf intestinal phosphatase (CIP) after electrotransfer onto PVDF membranes following SDS-PAGE. SW480 cells were transiently transfected with or without Src kinase for 24 h. Cell lysates were collected and subjected to SDS-PAGE. Following the eletrotransfer of the separated protein onto PVDF membranes, the membranes were incubated with CIP (100U/ml) in reaction buffer at 37°C overnight, then subjected to immunoblotting with indicated antibodies. **(E) Competition by pY232 and pY291 peptides additionally confirmed the site-specificity of the antibodies against pY232 and pY291 Fas.** This is based on the loss of pY232 and pY291 detection upon the competition with pY232 and pY291, respectively. Lysate of SW480 cells transiently transfected with Src kinase was subjected to SDS-PAGE and immunoblotting with indicated antibodies in the presence of indicated peptides. Unphosphorylated peptides (Y232 and Y291) served as controls (nonbinding peptides), and phosphorylated peptides (pY232 and pY291) served as peptides that blocked specific binding of the anti-pY232 and anti-pY291 antibodies to pY232 and pY291 of Fas, respectively.
(TIF)

S12 Fig Y232 and Y291 residues of Fas can be phosphorylated by SFKs, Src and Yes-1. Phospho-protein mobility shift detection that separated phospho-proteins based on their charges showed that overexpressing Src (left panels) or Yes-1 (right panels) in SW480 cells increased pY232 and pY291 Fas levels. SW480 cells were transiently transfected with control vector, V5-tagged Src, or V5-tagged Yes-1. Cell lysates were subjected to phospho-protein mobility shift (Phos-Tag) SDS-PAGE and immunoblotting with indicated antibodies.
(TIF)

S1 Text Supplementary Information. Lists of materials and methods, information concerning rectal cancer biopsies, and accession numbers of proteins referred to in the main text.
(DOCX)

## Abbreviations

ALPS: autoimmune lymphoproliferative syndrome
CDE: clathrin-dependent endocytosis
CIE: clathrin-independent endocytosis
CIP: calf intestinal phosphatase
DED: death-effector domain
DISC: death-inducing signalling complex
FasL: Fas ligand
hFas: human Fas
FADD: Fas-associated protein with death domain
LL: dileucine
PI: propidium iodine
PTPiI: protein tyrosine phosphatase inhibitor I
pY: phospho-tyrosine
pY232: phospho-Y232
RT: radiotherapy
sFasL: soluble FasL
SFK: Src family kinase
TNF: tumor necrosis factor
