# Supplementary figures and images for: An Evolution-Guided Analysis Reveals a Multi-Signaling Regulation of Fas by Tyrosine Phosphorylation and its Implication in Human Cancers
Source: PLoS Biol. 2016 Mar 4;14(3):e1002401. doi: 10.1371/journal.pbio.1002401 (PMC4778973; doi:10.1371/journal.pbio.1002401)

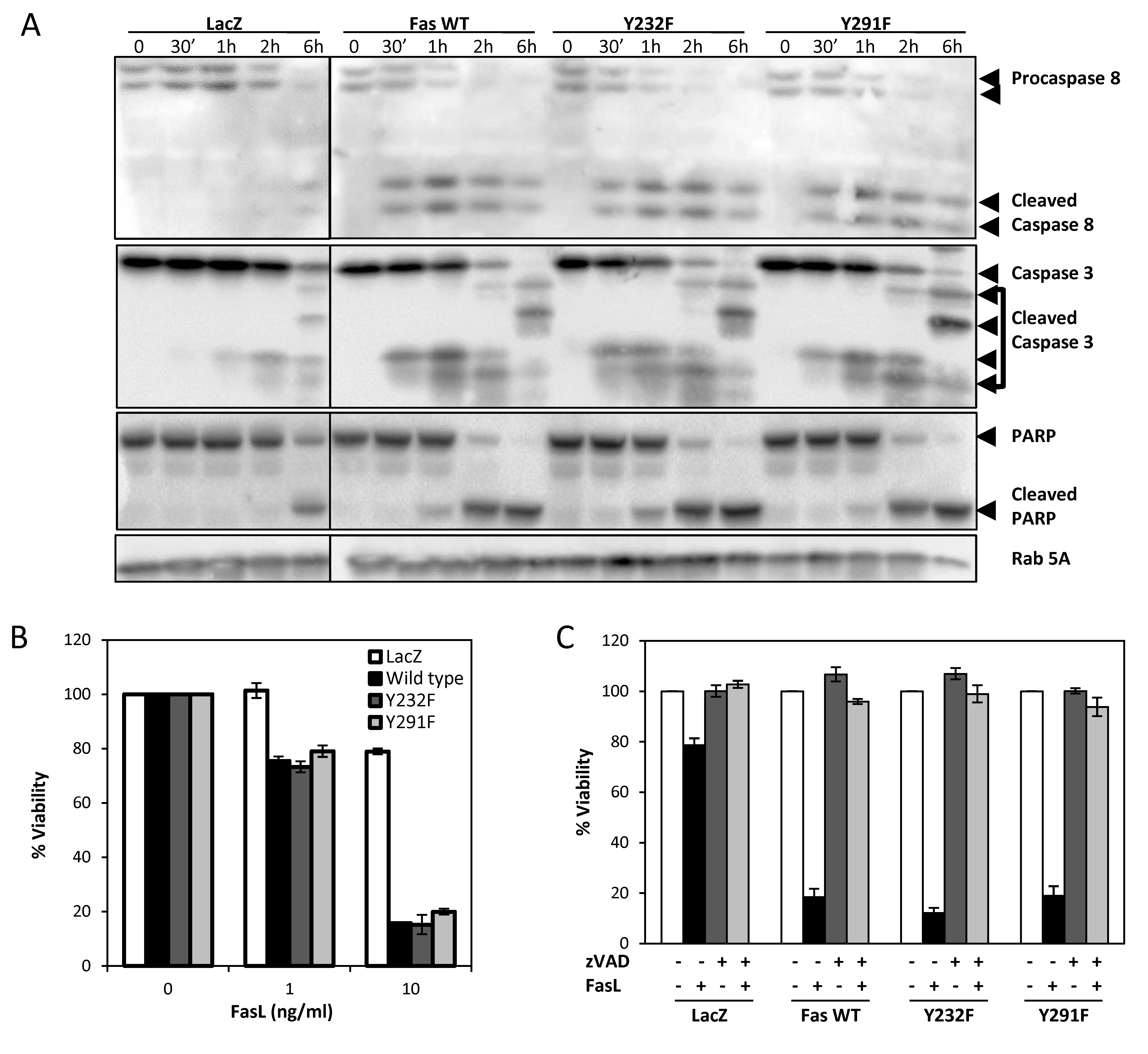

Supplement: S1 Fig — (A) SW480 cells stably expressing control protein (LacZ.V5) or indicated Fas.V5 proteins were treated with 10 ng/ml FasL crosslinked with anti-FLAG (M2) for indicated times, and cells lysates were collected and subjected to SDS-PAGE and immunoblotting with indicated antibodies. Initiation and execution of apoptosis is signified by the cleavage of caspase-8, caspase-3, and PARP. (B) Viability of cells treated with indicated doses of crosslinked FasL for 24 h was measured by WST-1 assay and presented as percentage of cell viability compared to untreated control cells. (C) Cells were pre-incubated with pan-caspase inhibitor, zVAD (10 μM), or DMSO for 30 min before incubating for 24 h with or without 10 ng/ml FasL crosslinked with M2 and assessed for cell viability using WST-1 assay. Note that the Y232F and Y291F mutations exhibit the same ability as wild-type Fas to transduce apoptosis in SW480 cells upon stimulation with crosslinked FasL, as shown by the activation of the caspase cascade and PARP cleavage (A) and cytotoxic assay (B). Similar to wild-type Fas, the cell death in cells carrying Y232F and Y291F mutants could be inhibited by the pan-caspase inhibitor zVAD, confirming the caspase-dependent apoptosis transduced by these unphosphorylated mutants (C). Numerical values underlying the data summary displayed in this figure can be found in S1 Data. (TIF) [file pbio.1002401.s002.tif]

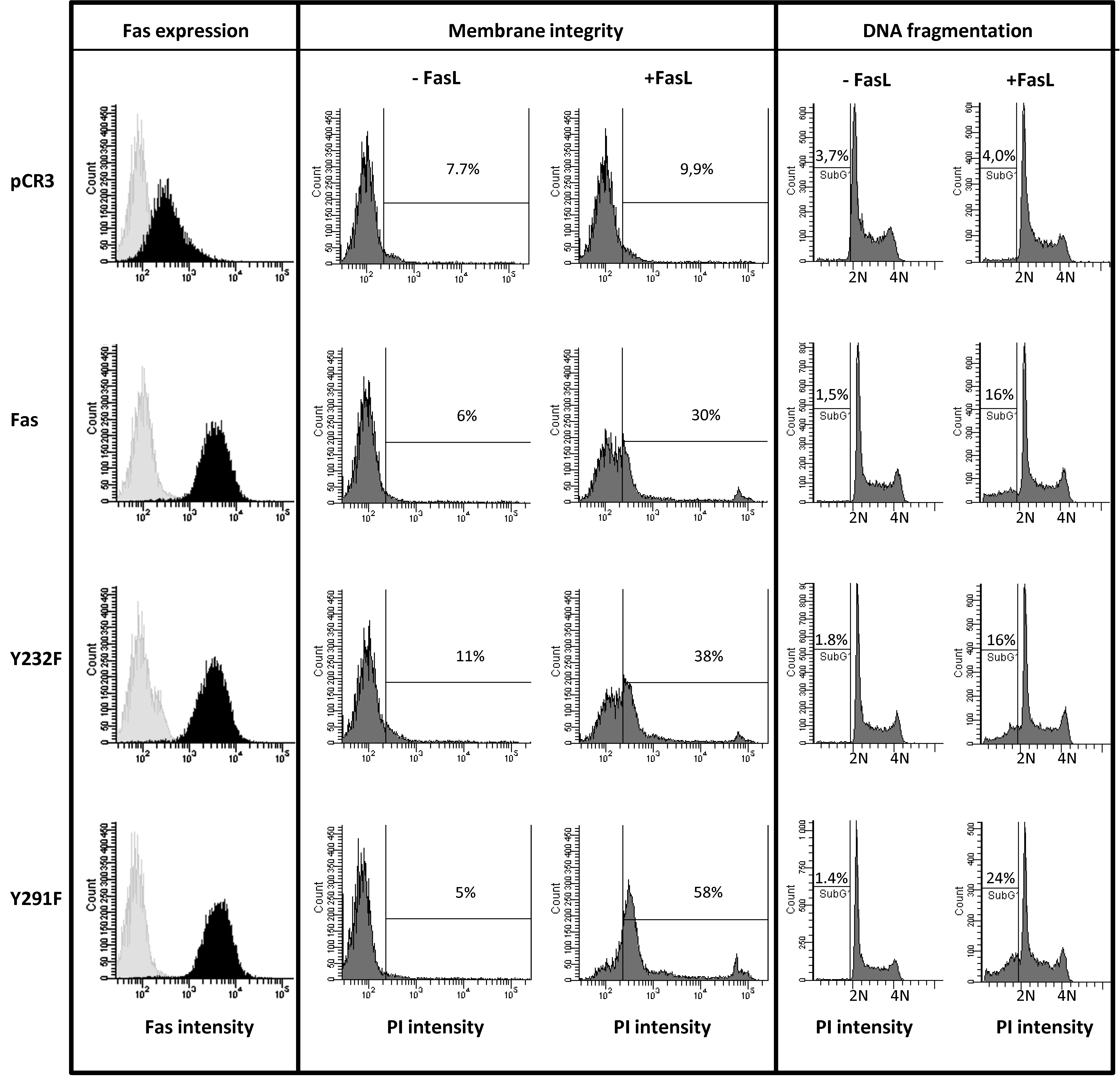

Supplement: S2 Fig — Left panel: flow cytometry analysis of Fas surface expression of WSU cells stably overexpressing control vector (pCR3), Fas, and unphosphorylated mutant Fas proteins (gray, isotype control; black, anti-Fas antibody). Middle panel: flow cytometry analysis of cell death assessed by the loss of plasma membrane integrity (PI staining without cell fixation) without or with activation with 50 ng/ml FasL crosslinked with 1 μg/ml anti-Flag (M2) for 20 h. Numbers shown are percentages of cells with positive staining for PI, representing dead cells. Right panel: flow cytometry analysis of apoptosis based on DNA fragmentation as assessed by the appearance of subG1 cell population (PI staining after cell fixation) without or with activation with 50 ng/ml FasL crosslinked with 1 μg/ml anti-Flag (M2) for 6 h. Numbers indicate percentage of cells that underwent apoptosis, having subG1 DNA content due to DNA fragmentation. Like in SW480 cells, the Y232F and Y291F mutations exhibited the same ability as wild-type Fas to transduce apoptosis in WSU cells upon stimulation with crosslinked FasL. (TIF) [file pbio.1002401.s003.tif]

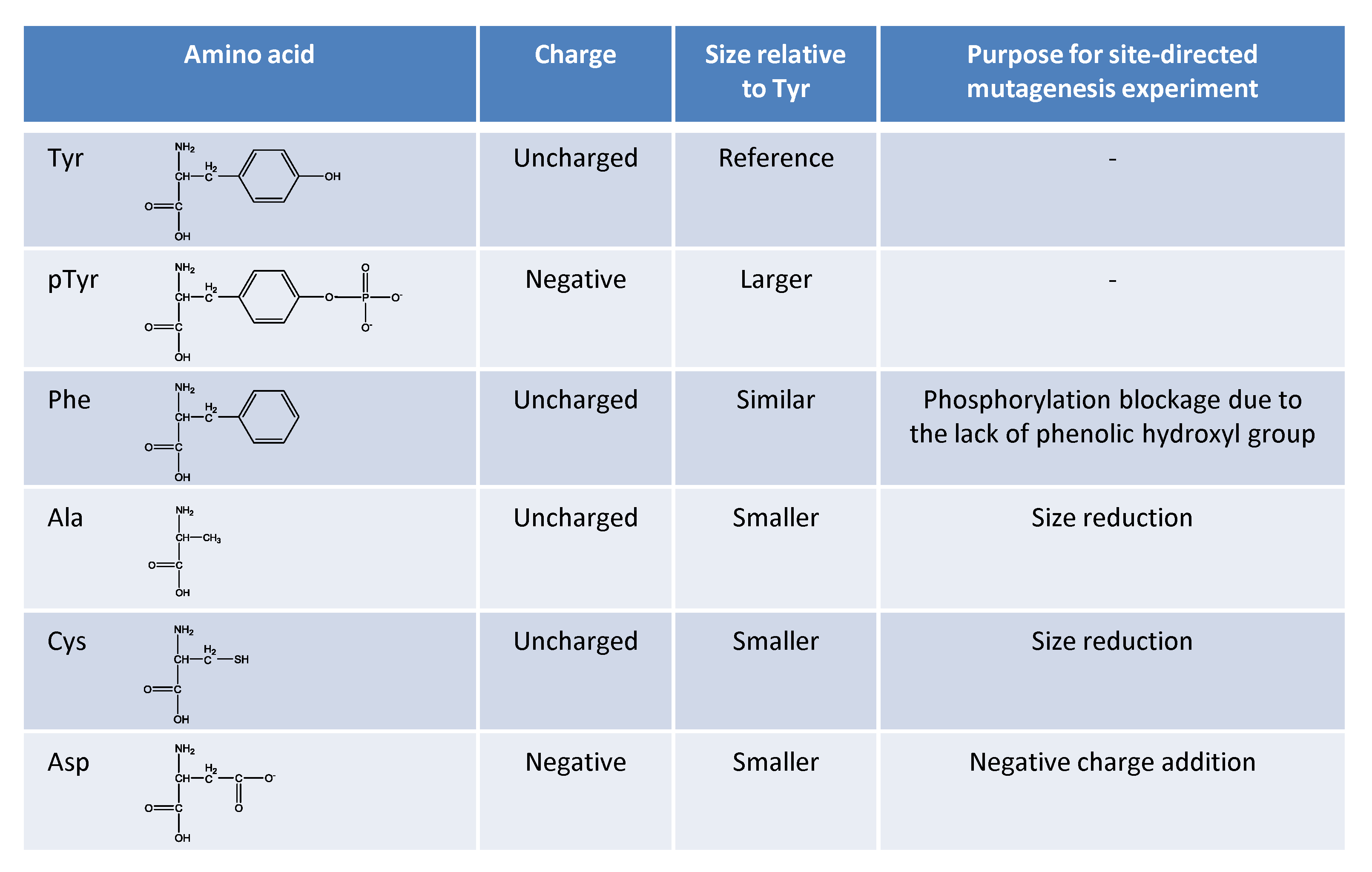

Supplement: S3 Fig — (TIF) [file pbio.1002401.s004.tif]

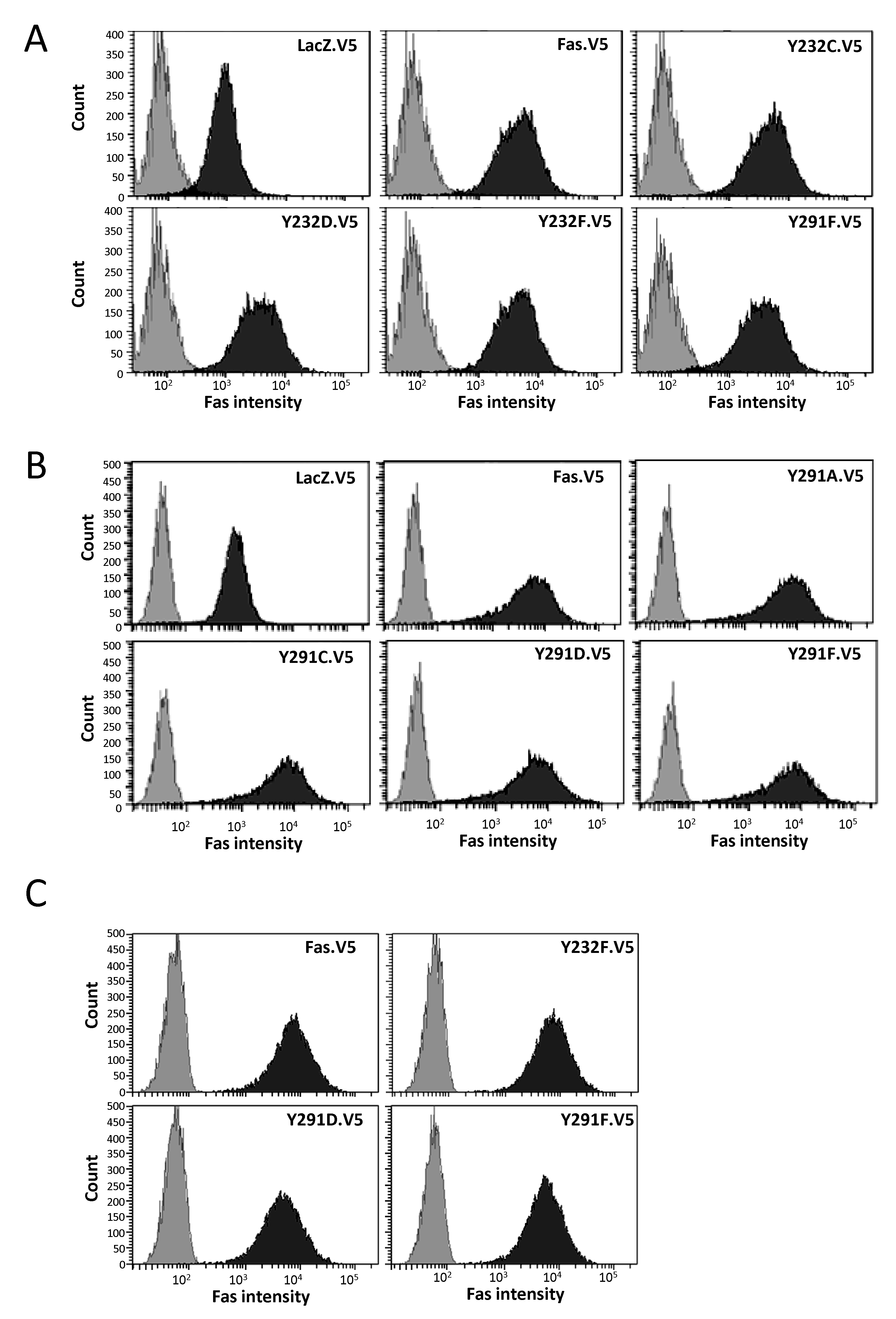

Supplement: S4 Fig — Flow cytometry analysis showing equivalent levels of Fas surface expression of SW480 cells stably overexpressing V5-tagged proteins (A) and (B), and SW480 cells stably over-expressing Fas V5-tagged proteins carrying silent mutations at the site targeted by a Fas siRNA (C) (gray, isotype control; black, anti-Fas antibody). (TIF) [file pbio.1002401.s005.tif]

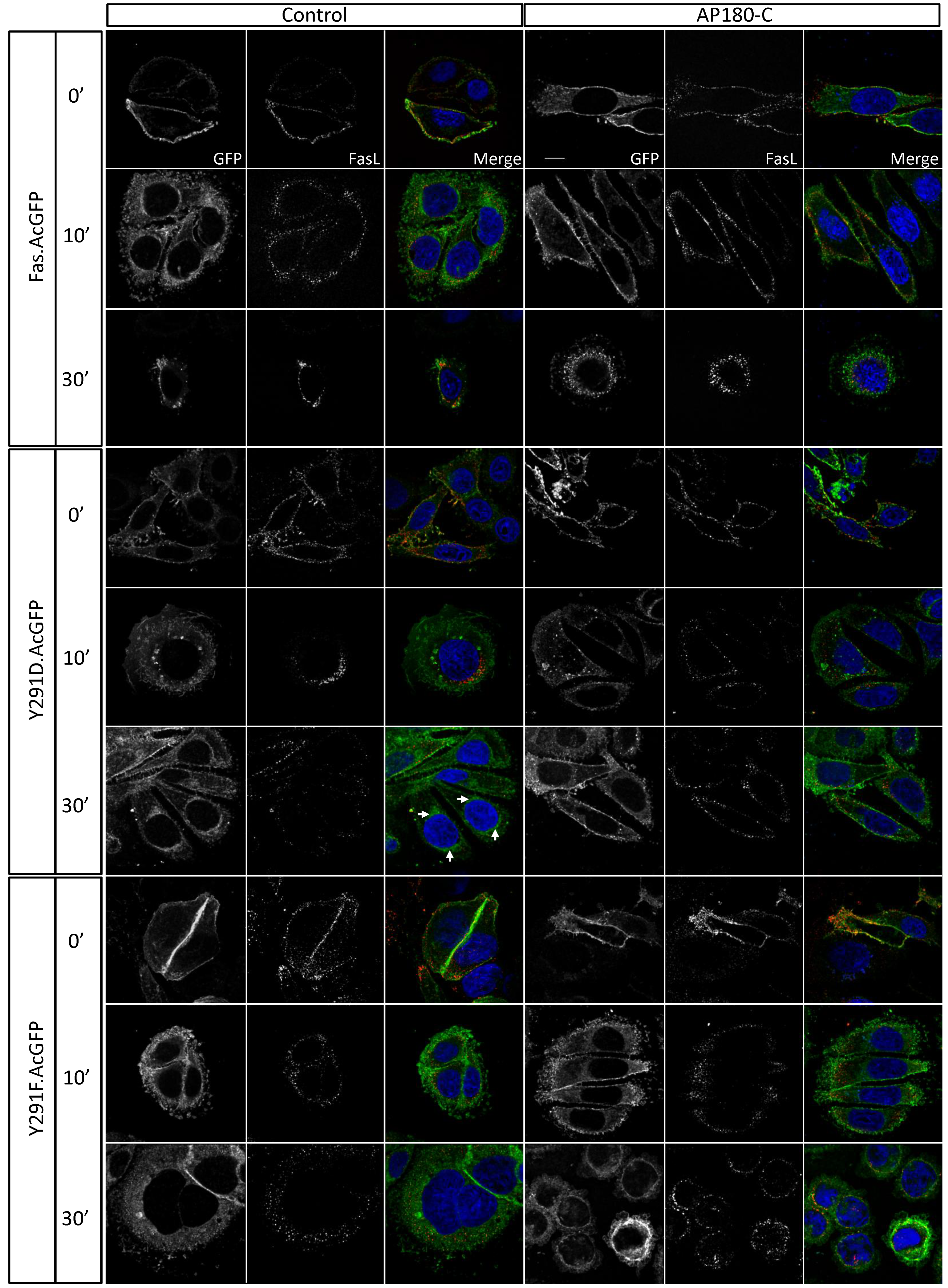

Supplement: S5 Fig — Supplementing the results presented in Fig 4B, unmerged images of SW480 cells subjected to synchronized FasL internalization assay and imaged by spinning disk confocal microscope are shown here. Images from each channel are shown in gray scale. In merged images, green represents AcGFP; red, FasL. (TIF) [file pbio.1002401.s006.tif]

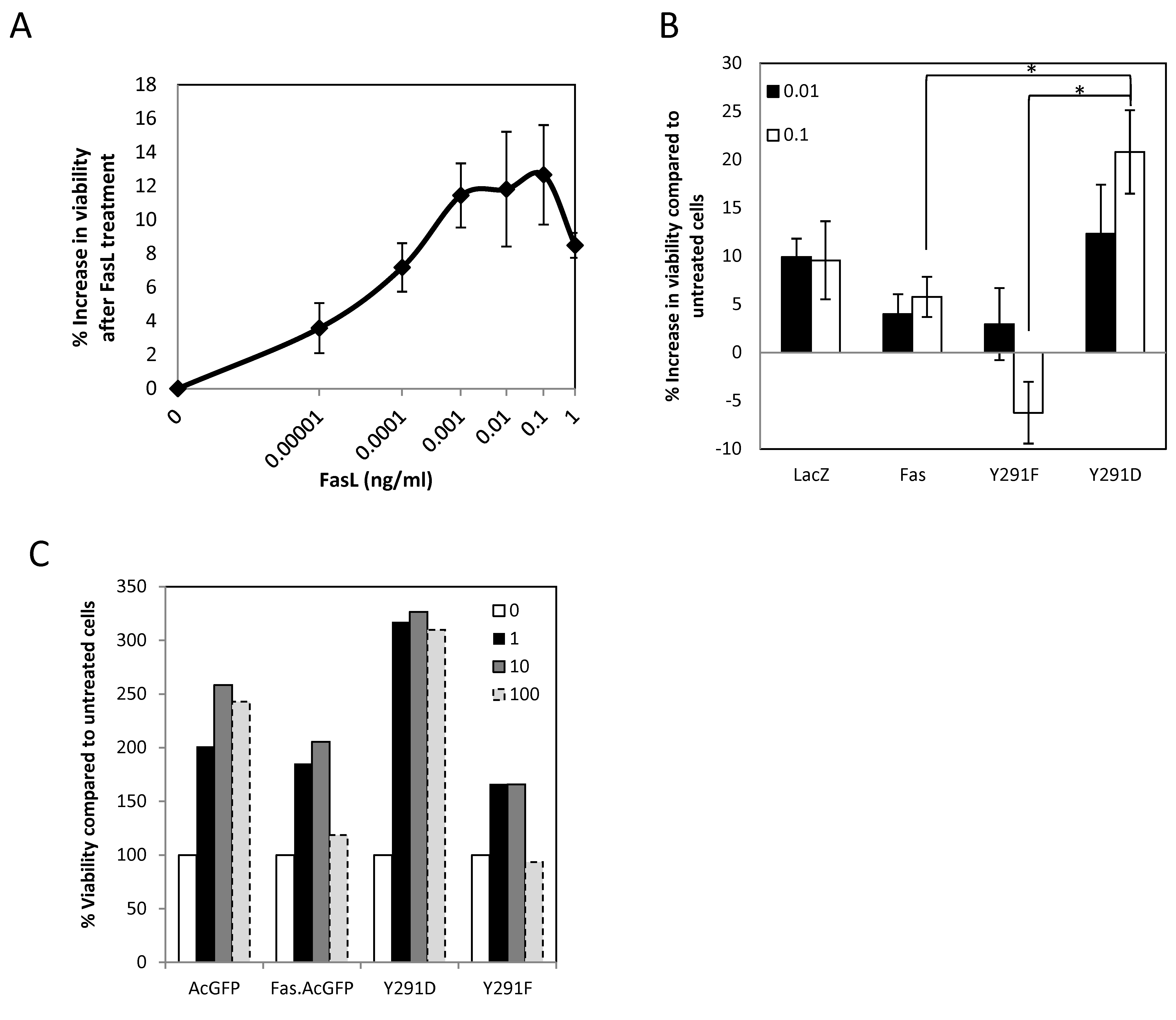

Supplement: S6 Fig — (A) Cell viability assay showing that FasL can activate cell proliferation. SW480 cells were synchronized to G1 phase by serum deprivation for 24 h and then left untreated or treated with indicated sublethal doses of soluble FasL (sFasL) for 48 h before viability measurement by WST-1 assay. (B) FasL-induced proliferation was enhanced in cells carrying Y291D Fas mutation. SW480 cells stably expressing V5-tagged LacZ or indicated Fas proteins were synchronized to G1 phase by serum deprivation in RPMI+0.1% BSA for 24 h and left untreated or treated with indicated concentration of uncrosslinked sFasL (ng/ml) for 48 h before viability measurement by WST-1 assay. Data is presented as the percent increase in cell viability after FasL treatment compared to untreated control cells. Values represent means ± SEM from at least three independent experiments (* p < 0.05, t test). (C) As in SW480 cells, the expression of Y291D in SW620 cells enhanced the proliferative effect of sFasL, whereas expression of Y291F Fas reduced the proliferative effect of sFasL. SW620 cells stably expressing AcGFP or indicated AcGFP-tagged Fas proteins were synchronized to G1 phase by serum deprivation and then treated with indicated concentration of uncrosslinked sFasL (ng/ml). Cell viability was measured using WST-1 assay. Numerical values underlying the data summary displayed in this figure can be found in S1 Data. (TIF) [file pbio.1002401.s007.tif]

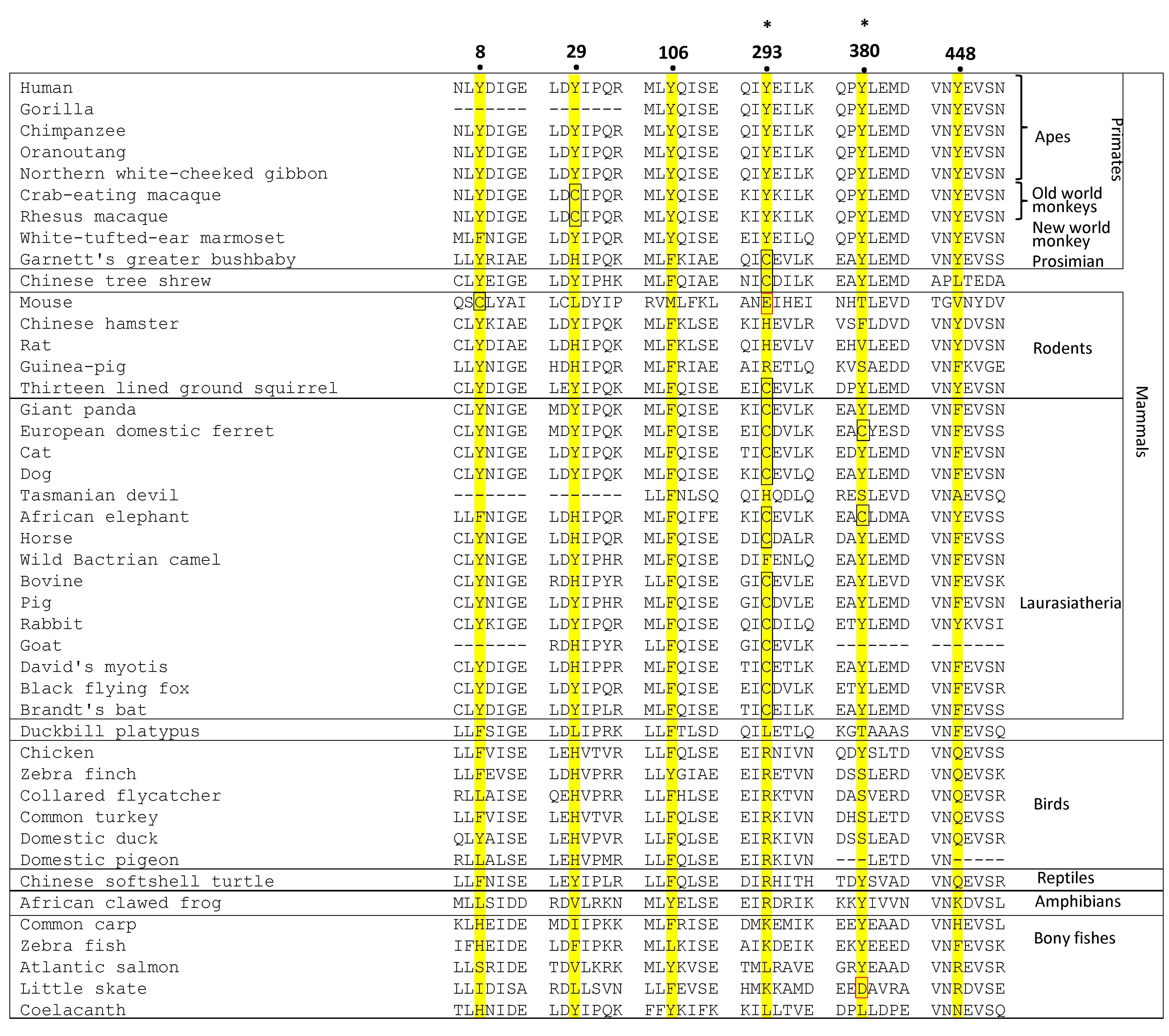

Supplement: S7 Fig — Partial alignment of sequences of caspase-8 from different vertebrates is shown. The amino acid positions of tyrosines in human caspase-8 are indicated (.). Only stretches of the sequence encompassing selected tyrosines are shown. The amino acid positions corresponding to tyrosines in human caspase-8 are highlighted. Cysteine and acidic amino acid residues at positions corresponding to the tyrosines of human caspase-8 are boxed in black and red, respectively (*, tyrosine phosphorylation sites: Y293 and Y380). (TIF) [file pbio.1002401.s008.tif]

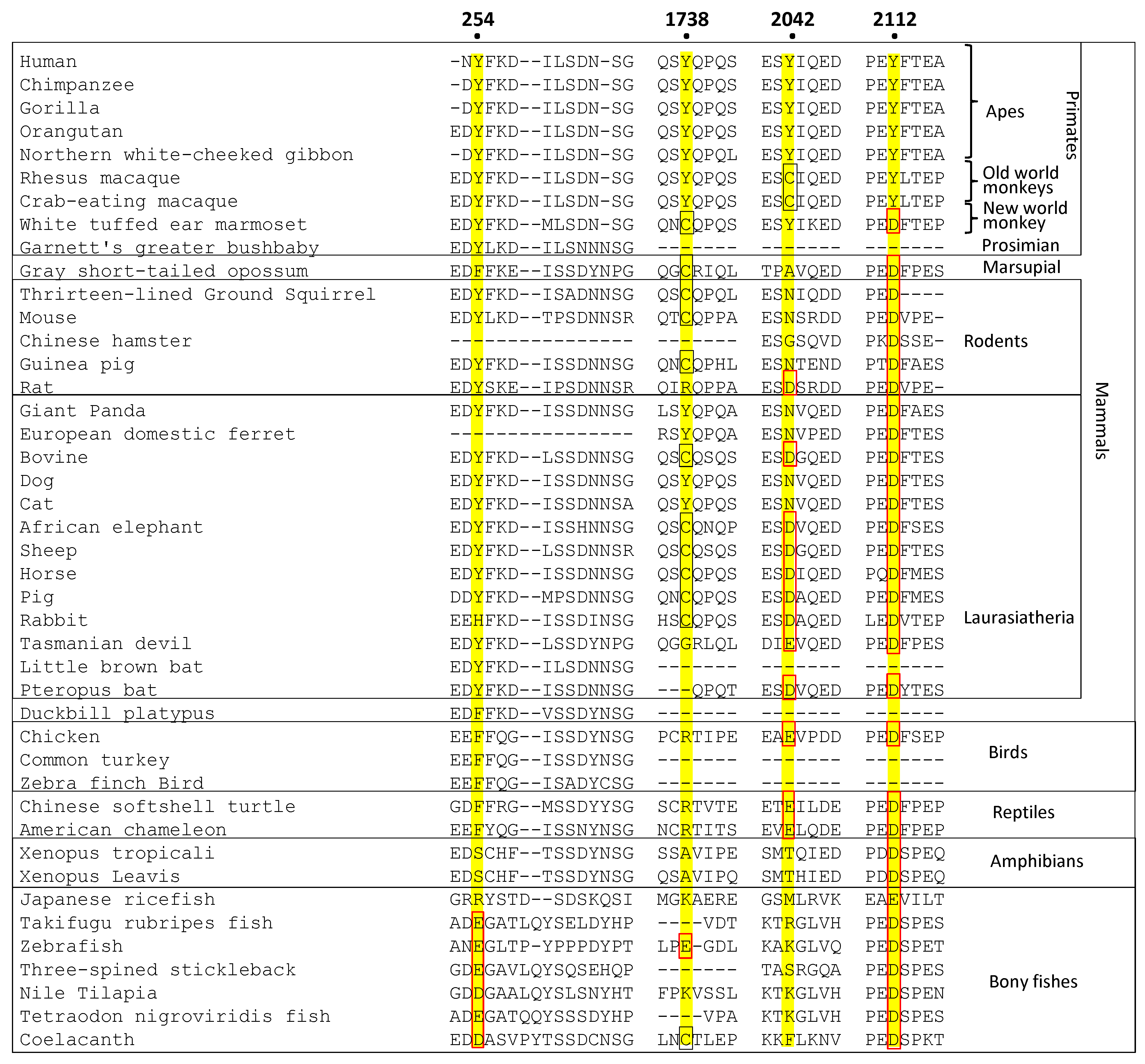

Supplement: S8 Fig — Partial alignment of sequences of FAP-1 from different vertebrates. The amino acid positions of tyrosines in human FAP-1 are indicated (.). Only stretches of the sequence encompassing selected tyrosines are shown. The amino acid positions corresponding to tyrosines in human FAP-1 are highlighted. Cysteine and acidic amino acid residues at positions corresponding to the tyrosines of human FAP-1 are boxed in black and red, respectively. (TIF) [file pbio.1002401.s009.tif]

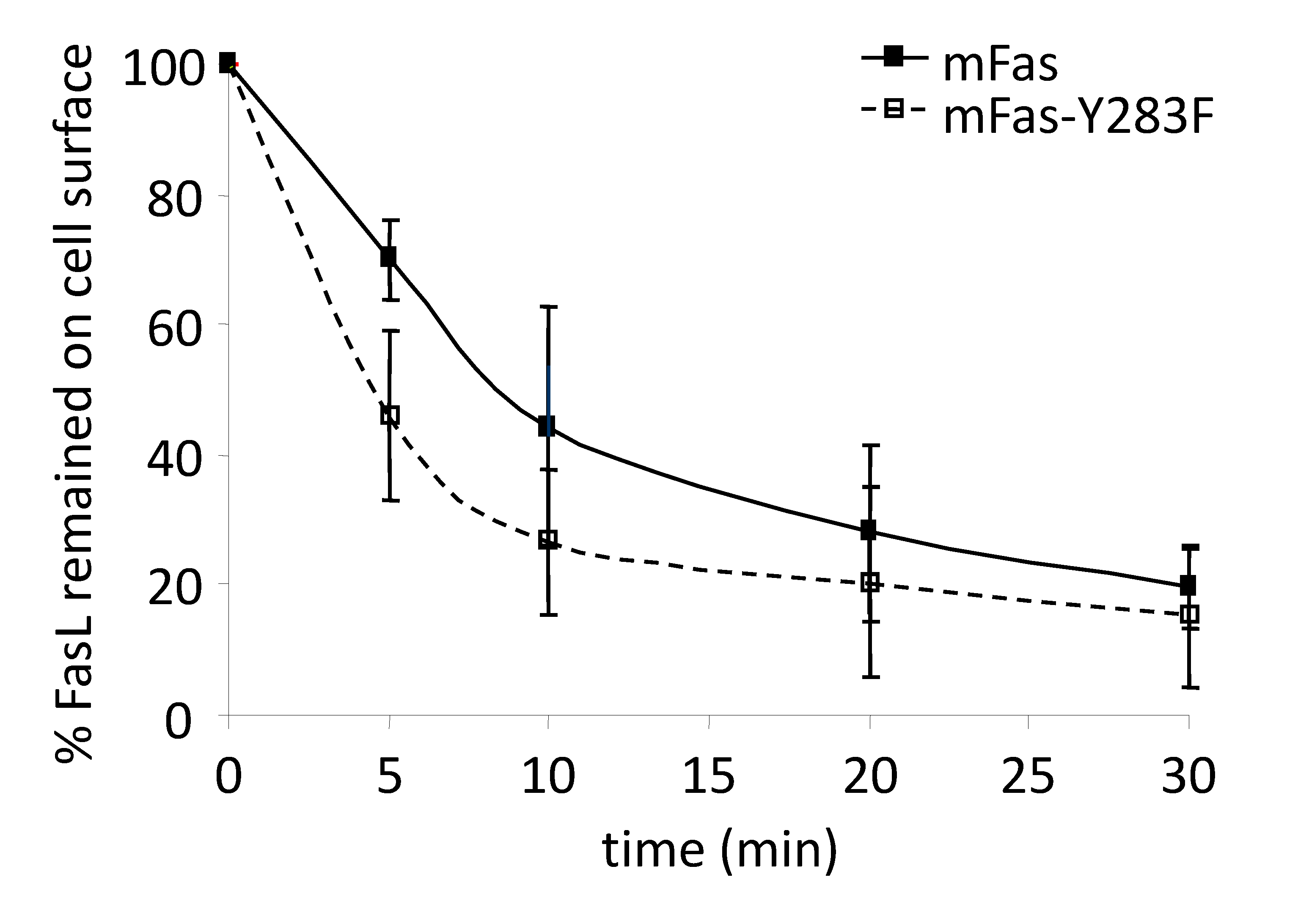

Supplement: S9 Fig — L1210 cells stably expressing wild type or Y283F murine Fas were subjected to synchronized internalization assay as described previously [22,43]. Briefly, crosslinked FasL was allowed to bind to the cell surface at 4°C before the internalization was triggered by raising the temperature to 37°C. The degree of Fas/FasL complex uptake was correlated with the downregulation of Fas/FasL complex from the cell surface by measuring the remaining FasL on the cell surface and normalized by the level of total FasL-bound before internalization was commenced. A lower percentage of FasL remaining on the cell surface indicates higher downregulation (internalization) of Fas/FasL complex. Numerical values underlying the data summary displayed in this figure can be found in S1 Data. (TIF) [file pbio.1002401.s010.tif]

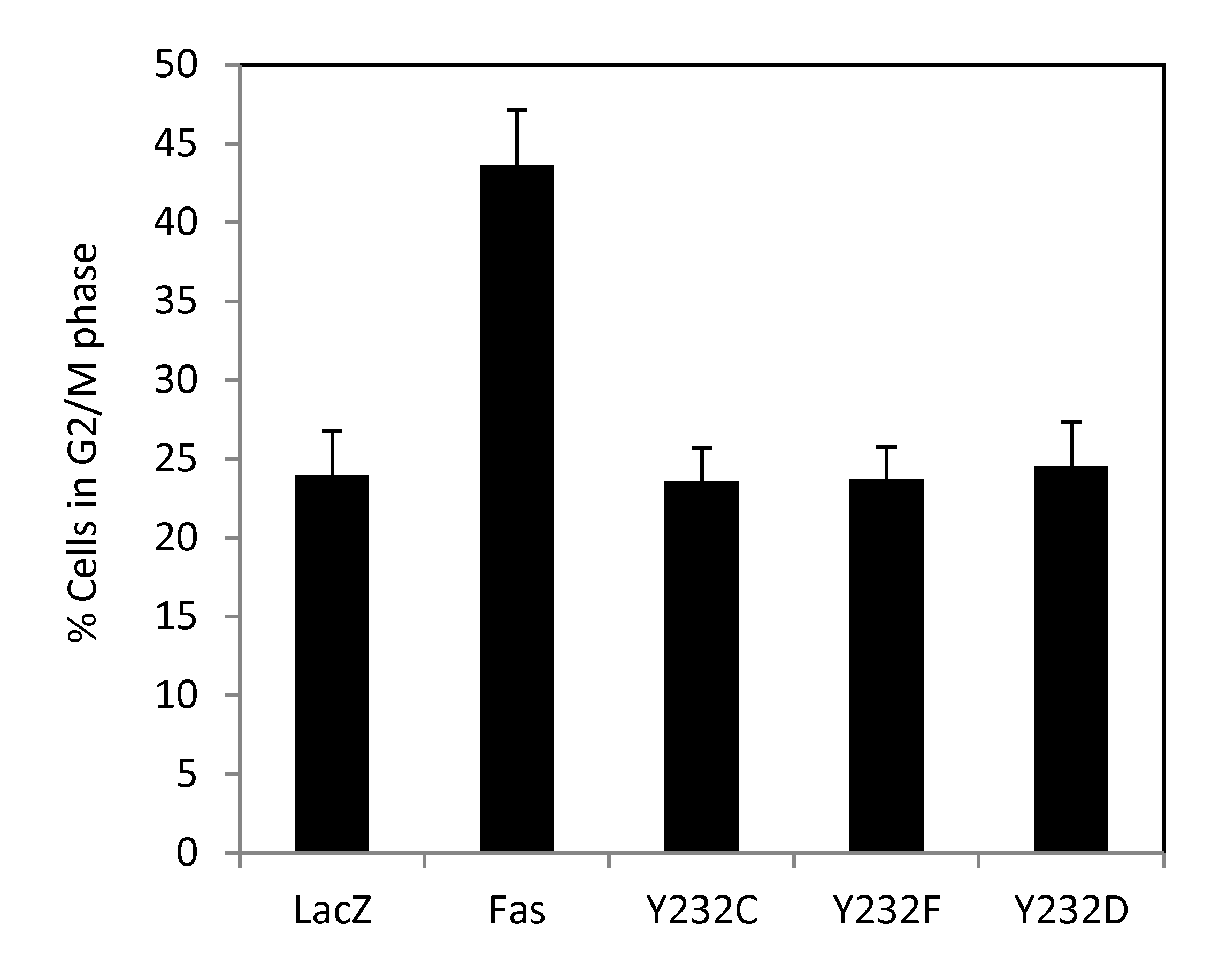

Supplement: S10 Fig — SW480 cells stably overexpressing V5-tagged Fas wild type and Y232 mutant proteins with equivalent Fas surface expression were subjected to cell cycle analysis based on DNA content. Data shown are mean ± SEM values of percent of cells in G2/M phase (DNA level = 4N) from three independent experiments. Numerical values underlying the data summary displayed in this figure can be found in S1 Data. (TIF) [file pbio.1002401.s011.tif]

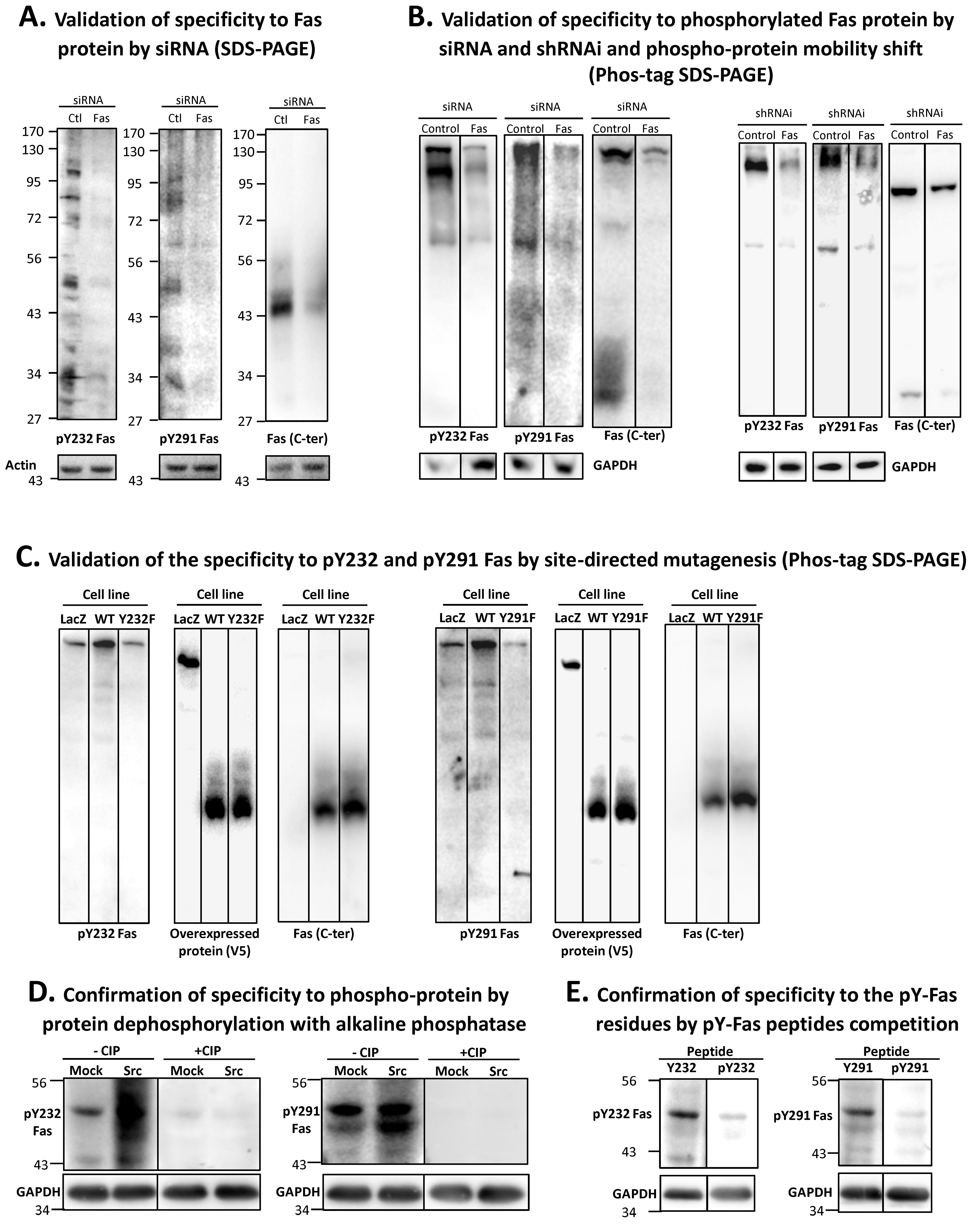

Supplement: S11 Fig — (A) Based on Fas siRNA, the antibodies against pY232 and pY291 Fas specifically detect Fas protein. Separation and detection of phosphorylated Fas protein in lysates from cells transfected with or without Fas siRNA by classical SDS-PAGE and immunoblotting demonstrating that pYFas antibodies detect Fas protein. SW480 cells were transiently transfected with 20 nM of control siRNA or siRNA directed against FAS gene. Cell lysates were then collected after 72 h of transfection and subjected to SDS-PAGE, where protein separation was based on molecular weights, followed by immunoblotting with indicated antibodies. Note a clear reduction in pY232 and pY291 Fas upon Fas silencing. (B) Based on siRNA and shRNA coupled with phospho-protein mobility shift SDS-PAGE, the antibodies against pY232 and pY291 Fas specifically detect the population of Fas protein that is phosphorylated. Mobility shift detection of pYFas protein in lysates from cells, with or without Fas Suppression by RNAi, demonstrating that the pYFas antibodies detect pYFas. SW480 cells were transiently transfected with 20 nM of control siRNA or siRNA directed against exon 3 of the FAS gene for 72 h (left panel) or stably infected with short hair-pin RNAi (shRNAi) against exon 9 of the FAS gene (right panel). Cell lysates were collected and subjected to mobility shift, phosphate affinity SDS-PAGE using acrylamide-pendant Phos-tag™, in which proteins were separated based on their degree of phosphorylation, followed by immunoblotting with indicated antibodies. Note that the antibodies against pY232 and pY291 detected slow-migrating Fas bands (upper part of the gel, characteristic of phosphorylated proteins), and the detection was clearly reduced upon the reduction of Fas protein by siRNA or shRNAi, confirming the specificity of the antibodies to phosphorylated Fas protein. (C) Based on site-directed mutagenesis, the antibodies against pY232 and pY291 Fas detect pY232 and pY291 residues in a site specific manner. Left [file pbio.1002401.s012.tif]

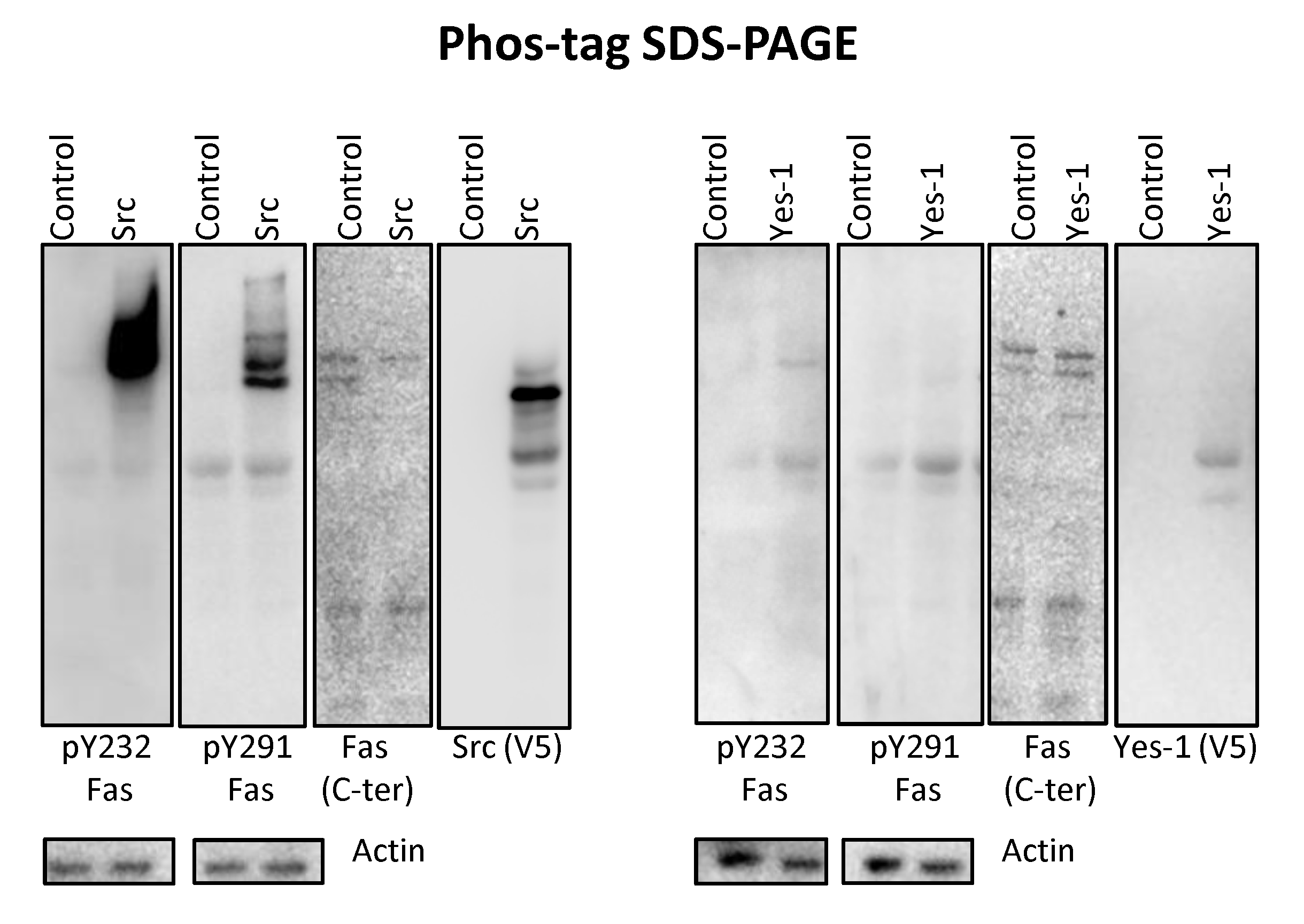

Supplement: S12 Fig — Phospho-protein mobility shift detection that separated phospho-proteins based on their charges showed that overexpressing Src (left panels) or Yes-1 (right panels) in SW480 cells increased pY232 and pY291 Fas levels. SW480 cells were transiently transfected with control vector, V5-tagged Src, or V5-tagged Yes-1. Cell lysates were subjected to phospho-protein mobility shift (Phos-Tag) SDS-PAGE and immunoblotting with indicated antibodies. (TIF) [file pbio.1002401.s013.tif]
