# Supplementary material for: An Evolution-Guided Analysis Reveals a Multi-Signaling Regulation of Fas by Tyrosine Phosphorylation and its Implication in Human Cancers
Source: PLoS Biol. 2016 Mar 4;14(3):e1002401. doi: 10.1371/journal.pbio.1002401 (PMC4778973; doi:10.1371/journal.pbio.1002401)
Supplement: S1 Text — Lists of materials and methods, information concerning rectal cancer biopsies, and accession numbers of proteins referred to in the main text. (DOCX) [file pbio.1002401.s014.docx]

**SUPPLEMENTARY INFORMATION**

**MATERIALS**

**Cell lines**

Colon cell lines CCD-18Co, SW480, and SW620 were purchased from the American Type Culture Collection (ATCC) and DLD-1, HCT15, HCT116, and HT29 from Leibniz-Institut, German collection of microorganisms and cell culture (DSMZ). All cells were maintained in PRMI 1640+Glutamax I (Gibco) supplemented with 10% fetal bovine serum (FBS) and maintained at 37°C, 5% CO_2_, except CCD-18Co cells, which were maintained in DMEM+Glutamax I+glucose (4.5g/L)+pyruvate (Gibco) supplemented with 1 0 % and 0.1 mM non-essential amino acids**.** For site-directed mutagenesis studies cell lines were established by transducing cells with lentivirus vector (pLenti6) carrying C-terminally V5- or AcGFP-tagged wild type Fas protein or Fas with one of the following mutations: Y232C, Y232D, Y232F, Y291A, Y291C, Y291D, Y291F, Y232F/Y291D, Y232D/Y291F, and Y232F/Y291F. Where indicated, cells were established by transducing cells with lentivirus vector (pLenti6) carrying C-terminally V5-tagged wild type Fas protein or Fas with one of the following mutations: Y232F, Y291D, Y291F, as well as silent mutations C619T and C622A (according to the mRNA sequence, accession no. NM_000043) which is located in the target site of Fas siRNA used in this study. As controls, cells were transduced with pLenti6-LacZ.V5 or pLenti6-AcGFP. Cells stably expressing the proteins of interest were selected by blasticidin resistance. For stably Fas knockdown cells, SW480 cells were transducing cells with lentivirus vector (pLVTHM-Blasticidin) carrying scrambled (non-targeted) or Fas shRNAi (see below) and cells stably expressing the RNAi were selected by blasticidin resistance.

**Antibodies and reagents**

The source of commercial antibodies and reagents are as followed: anti-FADD (Calbiochem); anti-caspase 9 (5B4, MBL); anti-caspase-8 (Alexis corporation);anti-caspase 3 and anti-PARP (Cell Signaling Technology), anti-Src, anti-Yes (R&D Systems); anti-human Fas (C-terminus) (C20), anti-SHP-1 anti-Rab5A (Santa Cruz); FITC-conjugated mouse anti-Fas (DX2, Miltenyi), horseradish peroxidase conjugated anti-rabbit and anti-mouse (Jackson ImmunoResearch); anti-mouse IgG-Alexa Flour 488, 546, and 647, anti-rat IgG-Alexa Fluor 488, anti-rabbit IgG-Alexa Fluor 546 (Life Technology), anti-BrdU (BD Bioscience), anti-Fas (Apo1-3, Alexis Corporation). Mouse anti-Flag (M2) from Sigma was used for FasL cross-linking. Recombinant human FasL (rhFasL) was from Alexis Corporation; dynasore, from Sigma; protein G sepharose beads from Zymed Laboratories, WST-1 from Interchim. Lipofectamine RNAiMAX reagent (Invitrogen) was used for siRNA transfection according to manufacturer's protocol for reverse transfection. JetPRIME reagent (Polyplus transfection) was used for transient DNA transfections. PP2, PP3, and PTPiI were from Calbiochem, dynasore from Sigma. Phostag acrylamide was from Wako Chemicals.

**siRNA:**

Src, Yes, and, SHP-1 siRNAs were from QIAGEN (catalog # SI02223921, SI00302218, and SI02658726, respectively), negative control siRNA (Luciferase) (5'-CGUACGCGGAAUACUUCGA-3') and Fas siRNA (Exon 3: 5'-AAGGAGUACACAGACAAAGCC-3') from Eurofins.

**Plasmids**:

Human Fas cDNA insert from pCR3-hFas (PS345) (a kind gift from P Schneider) was subcloned into pLenti6-V5 plasmid. All substitutions for Y232 and Y291 were performed using the Phusion site-directed mutagenesis kit (Life Technologies). pLenti6-AcGFP1 construct was generated by subcloning AcGFP1 insert from pLVX AcGFP1-N1 construct (Clontech) into pLenti6 vector. All constructs for Fas (wild type and mutants) tagged at the C-terminal with AcGFP were generated by subcloning Fas inserts from pLenti6-V5 constructs into pLenti6-AcGFP1 vector. The following plasmids were purchased from Addgene (plasmid numbers are given in parentheses): pDONR223-SRC [1] (23934), pDONR223-YES1 [1] (23938), pBABE-puro SHP1 WT (8575). Src, and Yes-1 inserts were subcloned into pLenti6-V5 plasmid. pCMVmyc-AP180-C was a gift from B. Nichols (LMB, Cambridge, UK). For shRNAi, scrambled (non-targeted) shRNAi (5'-CCTAAGGTTAAGTCGCCCTCG-3') and Fas shRNAi (Exon 9: 5'- GAAGCGTATGACACATTGA-3') were cloned into pLVTHM vector (Addgene Plasmid #12247[2]) whose GFP gene was previously removed and into which blasticidin resistance gene was cloned.

**Monoclonal antibodies against human pY232 and pY291 Fas**

The anti-pY232 Fas antibody clone (1C7.3) and anti-pY291 Fas (clone 33A9.2) are monoclonal antibodies of isotype G (IgG) that were produced in rat against synthesized phosphopeptide derived from amino acid sequence encompassing Y232 and Y291 site of human Fas (TNFRSF6, UniProt ID: P25445). They were affinity-purified anti-pY232 and pY291 and validated (Fig. S10). The production and validation of the antibodies are described below:

Hybridoma production**:** The hybridoma production was carried out following standard monoclonal antibody protocol. Briefly, the peptide, H-NLSDVDLSK(pY)ITTIAGVMC-OH (for anti-pY232) or H-HQLHGKKEA(pY)DTLIKDLKKA-OH (for pY291 antibody) (pY=phosphotyrosine) was coupled to a carrier protein (chicken ovalbumin) and immunized in Wistar rat with the coupled peptide emulsified in Freund's Complete Adjuvant (CFA) by intraperitoneal (IP) injection (Day 1). Second immunization was carried out on Day 15, followed by the challenges on Days 25, 26 and 27. The fusion of rat spleen with myeloma cell line was performed on Day 28. Subsequently, preclone screening for antigen specific clones was carried out. Positive clones were expanded and rescreened by immunoblotting using hybridoma culture supernatant, then fusion positive clones were subcloned and screen for antigen specific positive clones by ELISA.

Antibody purification: Selected hybridoma were maintained in Dulbecco's Modified Eagle Medium (DMEM) supplemented with 10% fetal bovine serum at 37°C, 5% CO2. Cells were separated from the supernatant by centrifugation. The antibody was purified by two steps of chromatography purification. 1. *Affinity purification by protein G*. The filtered supernatant was loaded onto a HiTrap protein G HP column (GE Healthcare). After washing the column, the bound IgG was eluted from the column by 0.1 M glycine-HCl, pH 2.7 and the buffer was exchanged to 20 mM sodium phosphate, pH 7.0 for application to the next purification step. 2. *Affinity purification by anti-rat IgG antibody*. The IgG purified by protein G column was applied to Hi-Trap NHS-activated HP column (GE Healthcare) conjugated with anti-rat IgG antibody. After washing the column, the bound Rat IgG was eluted from the column by 0.1 M glycine-HCl, pH 2.7 and the buffer was exchanged to phosphate buffered saline (PBS) pH 7.4 and stored at -20°C

Antibody validation: The antibodies were extensively validated for their specificity by immunoblot analysis using several approaches. Data concerning antibody validation are presented in S11 Fig. The data demonstrate the following: (A)Based on Fas siRNA, the antibodies against pY232 and pY291 Fas specifically detect Fas protein, (B) Based on siRNA and shRNA coupled with phospho-protein mobility shift SDS-PAGE, the antibodies against pY232 and pY291 Fas specifically detect the population of Fas protein that is phosphorylated, (C) Based on site-directed mutagenesis, the antibodies against pY232 and pY291 Fas detect pY232 and pY291 residues in a site specific manner, (D) Dephosphorylation by Calf intestinal phosphatase additionally confirmed that both antibodies against pY232 and pY291 Fas detected phosphorylated proteins, and (E) Competition by pY232 and pY291 peptides additionally confirmed the site-specificity of the antibodies against pY232 and pY291 Fas.

These data demonstrate the specificity of anti-pY232 antibody (clone 1C7.3) to the pY232 Fas and anti-pY291 antibody (clone 33A9.2) to the pY291 Fas.

**Human cancer tissue lysates**

Matched human tumor/normal tissue lysate kits were obtained from Protein biotechnologies (catalog numbers: colon, T7-6X-1 and T7-6X-2; breast, T2-6X-1 and T2-6X-2; ovarian, T5-6X-1; cervical, T4-6X-1; lung, T1-6X-1). Information regarding the tissues including cancer stages and lysate preparation can be found at the supplier's website (http://www.proteinbiotechnologies.com/products-human-lysate-kits.html).

**Rectal tissue lysates**

Rectal tumor tissues were obtained from patients, with official consent, according to translation research protocol: 'Translational study of post-translational modifications of Fas receptor in rectal cancer' (Etude translationnelle des modifications post traductionnelles du Récepteur FAS dans les cancers du Rectum, CARFAS; N° ID-RCB:2011A00777-34) established with Centre Antoine Lacassagne (Nice, France). Rectal cancer patients (age ≥18 yrs) having rectal adenocarcinoma, with tumor that was situated in the middle or inferior rectum and accessible to rigid rectoscopy and digital rectal examination and not undergoing chemotherapy for progressive cancer, were subjected to either contact radiotherapy (30-45 Gy/session) or external radiotherapy (1.8-5 Gy/session). The tumor biopsies (3-5 mm in diameter) were taken during rectoscopy by biopsy forceps before (t0) and after (t1) the treatment by radiotherapy (1st and 2nd biopsy, respectively). The biopsies were snap-frozen in liquid nitrogen immediately after excision. The frozen biopsies were stored in the vapor phase of liquid nitrogen. To prepare the tissue lysates, the tumors were homogenized in lysis buffer (25 mM Tris-HCl, pH7.5, 150 mM NaCl, 1 mM EDTA, 5 mM Na_3_VO_4_, 10 mM NaF, 10 mM NaPP, 25 mM β-glycerophosphate, protease inhibitor cocktail, 1% Igepal CA-630) to obtain soluble proteins and centrifuged to clarify. The process was performed on ice and the lysates were stored at -80°C until further analysis. The information concerning the tumors are listed in Table S1.

**METHODS**

**Multiple amino acid sequence alignment**

Fas protein sequences were compiled from Uniprot (http://www.uniprot.org/) and the sequence alignment was performed using Clustal Omega (http://www.ebi.ac.uk/Tools/msa/clustalo/). The order of species is arranged to reflect the evolutional distance from human based on the vertebrate topology used at UCSC genome browser, placental mammal phylogenetic tree, and alternative topologies for Laurasiatheres found at http://genomewiki.ucsc.edu/. Protein sequence identifications are listed in Table S2.

**DNA fragmentation-based assay of apoptosis by sub-G1 analysis:** Cells were plated in RPMI+10% FBS (5x10^5^ cells/well) in 6-well plate 24 h before the treatment. Cell death was triggered by by incubating cells with Flag-tagged recombinant human FasL (rhFasL; Alexis) plus 1 µg/ml anti-Flag Ab (M2) (crosslinked FasL), at 37°C, 5% CO_2_ and incubated for a specified time. Floating cells were collected and adherent cells were detached by trypsinization. Both cell populations were pooled and collected following centrifugation. Cells were then fixed with 70% ethanol (-20°C), washed in 38 mM sodium citrate (pH 7.4), stained with propidium iodide in the presence of RNase A, and analyzed with a flow cytometer (LSRFortessa, Becton Dickinson). The proportion of apoptotic cells represented by the subG1 peak was presented as percent of cell death [3].

**WST-1 Viability assay:** For cell death assessment, cells were seeded in RPMI+10% FCS at 10^4^ cells/well in 96-well plate 24 h before the treatment. Cell death triggered by crosslinked-FasL was done by incubating cells with Flag-tagged recombinant human FasL (rhFasL; Alexis) plus 1 µg/ml anti-Flag Ab (M2), at 37°C, 5% CO_2_ and incubated for a specified time. Where indicated, cells were pretreated with inhibitors 30 minutes prior to the addition of FasL. Following the incubation WST-1 reagent was added to each well. Cells were incubated for 4h before measurement at 450 nm and 690 nm (reference) with a spectrometer (Biotek). After subtracting blank control, absorbance at 690 nm was subtracted from that obtained at 450 nm. Viability of the cells was calculate as percentage of cell viability compared to control (untreated cells) using the following equation: % Viability = 100x (A450-A690)_treated_/(A450-A690)_control_, A, absorbance. For proliferation assays cells were seeded in RPMI+10% FBS at 5x10^3^ cells/well in 96-well plate for 24h. Cells were then washed with RPMI+0.1% BSA and synchronized by serum deprivation in RPMI+0.1% BSA for 24h before treatment with indicated reagent for 48h. Following treatment with indicated reagent for a specified time, WST-1 reagent was added. The measurement and viability calculation was done as described above. The percentage of the increase in proliferation is calculated by subtracting 100 from the calculated % viability.

**Bromodeoxyuridine (BrdU) incorporation assay** (microplate-based):

SW480 cells in RPMI+10% FBS (5x10^3^/well) were seeded in 96-well, half-area plate (black, transparent bottom, Greiner). After 24h, cells were synchronized by serum starvation in RPMI+0.1% BSA for 24h before activating with indicated concentration of soluble FasL (ng/ml) for 30 minutes at 37°C and then pulsed with 10 μM BrdU for 60 min. Subsequently, medium was removed and the plate was dried at 60°C for 1h. Cells were then fixed with 70% EtOH at room temperature for 10 minutes. After rinsing with PBS, DNA was denature by 2N HCl for 10 minutes at 37°C and then neutralized with 0.1 M borate buffer, pH 8. The cells were then incubated with blocking buffer for 1h, stained with anti-BrdU antibody, washed, and stained by Alexa Fluor 488-conjugated donkey anti-mouse antibody. After final wash, the fluorescence intensity was measured using microplate reader (Synergy4, Biotek).

**Boyden chamber cell migration assay:**

SW480 cells, pre-labeled with DilC18(3)-DS dye (Molecular Probes) according to manufacturer's instruction, were resuspended in RPMI medium supplemented with 0.1% fatty acid-free BSA (assay medium) at the concentration of 2x10^5^ cells/ml. Fluoroblok inserts (Falcon) were placed in the wells of 24-well plates, each prefilled with 0.75 ml of assay medium without or with 1 ng/ml FasL. Cells (5x10^4^) were then added to the top chamber of the Fluoroblok membrane insert and allowed to migrate for 2 h at 37°C, 5% CO_2_. Cells where then fixed with 4% paraformaldehyde in PBS for 10 minutes at room temperature. The fluorescently labeled migrated cells on the underside of the inserts were imaged using a wide field inverted microscope with 10x objective (Nikon TiE). From 5 randomly taken images per insert, the number of cells migrated through the membrane pores were counted using Analyze Particles function in FIJI software[4].

**SDS-PAGE and immunoblotting:**

Unless otherwise indicated, cells were seeded in 10-cm dishes at 4x10^6^ cells/dish or in 6-well plate at 2.5x10^5^ cells/well in RPMI+10% FBS for 24h. For cells that were subjected to FasL treatment, they were then washed with RPMI+0.1% BSA and synchronized by serum deprivation in RPMI+0.1% BSA for 24h before treatment with indicated concentrations of FasL for 5 minutes. Floating cells were then placed in 15-ml conic-bottom tube (BD Falcon) and collected following a centrifugation. Meanwhile the lysis buffer (120 mM Tris-HCl pH 6.8, 4% SDS, 10 mM NaP-P, 10 mM NaF, 25 mM β-glycerol phosphate, 5 mM NaVO_4_, protease inhibitor cocktail), preheated at 95°C ('hot SDS' buffer) was rapidly added to the adherent cells that remained on the dish. The lysate was collected and pooled with the pellet of floating cell population and then heated at 95°C for 5 minutes. The lysate was stored at -80°C until analysed by SDS-PAGE and immunoblotting. For human tissue lysates, an equal volume of 2x hot SDS buffer were added to the lysate before proceeding to standard SDS-PAGE sample preparation.

*Classical SDS-PAGE and immunoblotting:* Standard SDS-PAGE with 10% acrylamide gels were performed. Subsequently, proteins were blotted onto PVDF membranes. The electrotransfer was carried out in Tris-Glycine transfer buffer overnight at 4°C. After the transfer, the membranes were quickly dipped in EtOH and then air dried. The membranes were then blocked with 4% non-fat dried milk in TBS+0.1% Tween for 1h before incubation with primary antibodies overnight at 4°C. After 3 washes with TBS+0.1% Tween, membranes were incubated with HRP-conjugated antibodies for 1h at room temperature. After 3 washes with TBS+0.1% Tween, membranes were incubated with Immobilon Western Chemiluminescent HRP Substrate (Millipore) and images were taken with Odyssey Fc imaging system (Licor). Bands detected at expected sizes of proteins of interest are presented. For phopho-Fas and the C-terminus of Fas (Fas C-ter), bands detected at expected sizes of canonical Fas monomers (between 43 and 56 kDa are presented) and where indicated, these bands were subjected to densitometric analysis using ImageStudio software (Licor) with background correction using median intensity of pixels surrounding the selected Fas bands.

*Phospho-protein mobility shift Phosphate affinity SDS-PAGE and immunoblotting:* The procedure was carried out according to manufacturer's suggestions with some modifications. SDS-PAGE with 7.5% poly acrylamide gels containing 10 µM acrylamide-pendant Phos-Tag (Wako) was performed. The maximum of 20 µg of proteins was loaded into each well of 15-well gel. Molecular weight standards were omitted as the proteins were not separate based on sizes but on charges. The proteins were allowed to migrate slowly at 60V. Once the migration terminated, gel were soaked in 10 mM EDTA for 15 minutes. Subsequently, proteins were blotted onto PVDF membranes. The electrotransfer was carried out in Tris-Glycine transfer buffer + 1 mM EDTA overnight at 4°C. After the transfer, membranes were subjected to the same treatment as membranes from classical SDS-PAGE.

**Cell surface protein expression analysis by FACS**

Cells were seeded in 6-well plate at 5x10^5^ cells/well in RPMI+10% FBS for 24h. After trypsinization and a wash with PBS cells were placed on ice and stained with antibodies against extracellular domains of Fas followed by the staining with corresponding fluorescence conjugated antibodies. Cells were then subjected to FACS analysis (LSRFortessa or FACSCalibur, Becton Dickinson).

**Coimmunoprecipitation**

The postnuclear supernatant (PNS) of cells (8x10^6^) stimulated or unstimulated, as indicated, was prepared by collecting cells in ice-cold lysis buffer (25mM HEPES, 150mM NaCl, 1mM EGTA, 10 mM NaP-P, 10 mM NaF, 25 mM β-glycerol phosphate, 5 mM NaVO_4_, protease inhibitors cocktail) containing 1% Nonidet P-40) followed by sonication and subsequently centrifugation to remove cell debris. The PNS was then incubated with protein G agarose beads conjugated with anti-Fas (APO-1-3) at 4°C for 18h. The beads were washed four times with lysis buffer and the immunoprecipitates were eluted from beads with Laemmli buffer at 95°C for 5 min, then subjected to SDS–PAGE, followed by immunoblotting.

**Supplementary Table S1. Rectal cancer biopsy information.**

| **ID** | **Stage** | **Radiotherapy treatment** | **Dose (Gy)** | **Days after treatment** |
| --- | --- | --- | --- | --- |
| **BCAL_30** | **T4** | **EBRT** | **18** | **7** |
| **BCAL_32** | **T2** | **CXB** | **30** | **13** |
| **BCAL_33** | **T3** | **EBRT** | **12** | **21** |
| **BCAL_34** | **T3** | **CXB** | **35** | **14** |
| **BCAL_38** | **T3** | **CXB** | **60** | **14** |
| **BCAL_39** | **T2** | **CXB** | **40** | **13** |
| **BCAL_40** | **In situ** | **CXB** | **30** | **14** |
| **BCAL_42** | **T3a** | **CXB** | **55** | **13** |
| **BCAL_41** | **T3b** | **CXB** | **30** | **19** |

*EBRT: External beam radiotherapy (concomitantly with chemotherapy: Cepecitabine),*

*CXB: contact X-Ray Brachytherapy* *50 Kv*

**Supplementary Table S2. Amino acid sequence accession numbers.**

**SUPPLEMENTARY REFERENCES:**

1. Johannessen CM, Boehm JS, Kim SY, Thomas SR, Wardwell L, Johnson LA, et al. COT drives resistance to RAF inhibition through MAP kinase pathway reactivation. Nature. 2010;468(7326):968-72. PubMed PMID: 21107320.

2. Wiznerowicz M, Trono D. Conditional suppression of cellular genes: lentivirus vector-mediated drug-inducible RNA interference. J Virol. 2003;77(16):8957-61. PubMed PMID: 12885912.

3. Chakrabandhu K, Herincs Z, Huault S, Dost B, Peng L, Conchonaud F, et al. Palmitoylation is required for efficient Fas cell death signaling. Embo J. 2007;26(1):209-20. Epub 2006 Dec 7.

4. Schindelin J, Arganda-Carreras I, Frise E, Kaynig V, Longair M, Pietzsch T, et al. Fiji: an open-source platform for biological-image analysis. Nat Meth. 2012;9(7):676-82. doi: http://www.nature.com/nmeth/journal/v9/n7/abs/nmeth.2019.html#supplementary-information.
